# Economic evaluation of primary health care integration and targeted mass drug administration in counties in western Kenya

**DOI:** 10.3389/frhs.2026.1858666

**Published:** 2026-09-22

**Authors:** Josphat Martin Muchangi, Wyckliff Omondi, Meshack Ndirangu, Carol Karutu, Irene Chami, Gilbert Wangalwa, Michael Ofire, Robert Ofwete, Kioko Kithuki, Nelly Saiti, Samuel Kamau, Lenson Kariuki, Paul Kibati, Japheth Athanasio, Geoffrey Njuhi, Yvonne Opanga, Emmanuel Musombi, Faith Mutegi, Dollycate N. Wanja, Festus Kipkorir, Hesbon Aoko, Chitiavi Juma

**Affiliations:** 1 Amref Health Africa, Nairobi, Kenya; 2 Vector-Borne & Neglected Tropical Diseases Unit, Ministry of Health, Nairobi, Kenya; 3 The END Fund, Nairobi, Kenya; 4 Reinit Research Limited, Nairobi, Kenya; 5 Department of Health Systems Strengthening, Ministry of Health, Nairobi, Kenya; 6 Children Investment Fund Foundation, Nairobi, Kenya

**Keywords:** cost effectiveness analysis, cost parameters, disability-adjusted life years (DALY), economic evaluation, incremental cost-effectiveness ration (ICER), Markov model, mass drug administration (MDA), soil-transmitted helminths (STH)

## Abstract

**Introduction:**

The 2025 World Health Organization (WHO) Global Report on Neglected Tropical Diseases (NTDs) highlights major progress made worldwide in controlling and eliminating these diseases. Sustaining NTD control requires cost-efficient strategies integrated into routine health systems. We evaluated the cost-effectiveness of integrating soil-transmitted helminths (STH) and schistosomiasis (SCH) treatment into routine Primary Health care (PHC) services compared with the campaign-based approach across four counties and selected sub-counties, wards, and primary health facilities in western Kenya. While the overall study assessed feasibility, treatment coverage, and economic outcomes, this manuscript evaluated the cost-effectiveness of integrating STH/SCH treatment into primary healthcare (PHC) versus campaign-based mass drug administration (MDA) in western Kenya.

**Methods:**

A quasi-experimental mixed-method study design was adopted for field-based data collection and analysis in 24 wards (12 wards for PHC-integrated, 12 wards for the campaign) across four counties. Cost data was collected from programme records. Cost parameters were informed by the WHO-CHOICE disease model, and relevant economic evaluation literature. A Markov state-transition model simulated a 5,000-person cohort over 5 years, estimating the Disability Adjusted Life Years (DALYs) averted using Global Burden of Diseases (GBD)disability weights (0.006 light, 0.024 moderate, 0.049 heavy infection). ICERs were calculated from a health system perspective. Study teams supporting both intervention arms received training to ensure consistent implementation and clear differentiation between the campaign and PHC-integrated delivery models.

**Results:**

The costing analysis showed that the campaign-based approach accounted for the largest share of program expenditures Kes 17.26 million (USD 133,814) against Kes 9.74 million (USD 75,519) for PHC integrated; a 44% reduction, largely due to its higher logistical and operational requirements, although it achieved the highest immediate treatment coverage. In contrast, the PHC-integrated model operated with lower overall costs, primarily associated with consumables and routine service delivery, while relying on existing health system infrastructure and staff including the Community health providers (CHPs). Campaign-based approach achieved higher coverage of 102.3% compared to 77.3% averting 19,230 DALYs compared to 12,840 for PHC integrated (additional 6,390 DALYs). This however translated to USD 0.25 (Kes 32.48) (cost per person treated (387,852 targeted Vs 299,960 treated) against USD 0.3 (Kes 39.54) per person treated (426,207 targeted against 436,059 treated) by the campaign approach. Economic modelling using a simulated cohort of 1,000 individuals annually over a five-year time horizon indicated that PHC integration could generate cost savings over time compared with repeated campaign delivery, highlighting its potential as a more sustainable approach for long-term disease control within routine health systems. Qualitative findings showed strong stakeholder support for PHC integration as a more sustainable and patient-centered approach than campaign-based MDA. Although MDA achieved higher coverage, PHC integration improved continuity of care through existing health system structures. Participants identified supply chain reliability, sustainable financing, digital reporting, supportive supervision, continuous capacity building, and county ownership as critical prerequisites for successful scale-up. Together, the two approaches treated 736,019 people, illustrating complementary strengths of scale versus cost efficiency. Given this evidence, our calculated ICER for campaign-based MDA, USD 9.13 per DALY averted demonstrates exceptional value for money, well below Kenya's likely willingness-to-pay threshold of about USD 2,000.

**Conclusion:**

Campaign MDA is extremely cost-effective for rapid, large-scale DALY reduction. PHC integration is more economical and sustainable for routine delivery. A hybrid approach with PHC as the backbone with periodic campaigns, optimizes both impact and sustainability. While PHC integration generated cost savings over time compared with repeated campaign approach, a cumulative 5-year DALY averted scaled to full cohort of 5,000 individuals indicates the campaign intervention averts 6,390 more DALYs than the PHC integration.

## Introduction

Neglected Tropical Diseases (NTDs) control and elimination, especially Soil-Transmitted Helminths (STH) and Schistosomiasis (SCH) remain global public health priorities as they continue to impact vulnerable populations in low- and middle-income economies where health systems face competing priorities. The 2025 World Health Organization (WHO) Global Report on Neglected Tropical Diseases (NTDs) highlights major progress made worldwide in controlling and eliminating these diseases (1). Several interventions have contributed to this progress, including preventive drug treatment, improved disease monitoring, better diagnostic tools, stronger supply systems, and increased collaboration between governments, pharmaceutical companies, development partners, and international organizations (1, 2).

As a result of these actions, the global burden of NTDs has declined significantly. Between 2015 and 2021, the number of disability-adjusted life years (DALYs) lost due to NTDs decreased from about 17.2 million to approximately 14.1 million. During the same period, NTD-related deaths dropped from an estimated 139,000 to around 119,000 deaths worldwide. The report also shows that the number of people affected by NTDs has fallen dramatically, from about 1.9 billion people in 1990 to just over 1 billion in 2021. In 2023 alone, approximately 1.495 billion people required interventions to prevent or treat NTDs, which represents 122 million fewer people compared with 2022 and a 32% reduction from the 2010 baseline (1). In terms of treatment coverage, 867.1 million people received treatment for at least one neglected tropical disease in 2023, and 99% of these individuals were treated through preventive chemotherapy, a strategy that involves giving safe medicines to populations at risk without prior diagnosis (1).

Sustaining the progress made in controlling neglected tropical diseases (NTDs) requires the adoption of cost-efficient and locally sustainable intervention strategies that can be maintained within national health systems without heavy reliance on external donor support. There is growing global consensus that long-term control and eventual elimination of helminth infections depend on integrating preventive chemotherapy and related NTD services into routine primary health care (PHC) systems, rather than continuing to depend solely on vertical mass treatment campaigns. The World Health Organization's NTD Roadmap 2021–2030 highlights integration of NTD services into primary health care and Universal Health Coverage (UHC) as a key strategy for strengthening health systems, improving sustainability, and increasing country ownership of NTD programmes (2, 3).

Emerging evidence from implementation research in several endemic countries supports this shift. Studies conducted in Ethiopia, Uganda, and Tanzania have demonstrated that integrating community-wide treatment for soil-transmitted helminths and schistosomiasis within PHC delivery platforms improves access to treatment, enhances equity in service coverage, and promotes program sustainability while reducing dependence on externally funded vertical campaigns (4–7). Such integrated approaches allow countries to leverage existing health infrastructure, community health workers, and routine health information systems, thereby lowering operational costs and enabling national health systems to sustain NTD control efforts over the long term (7).

Africa has benefited substantially from mass drug administration (MDA) campaigns targeting school-going children and, in some settings, entire communities or households as a strategy for controlling soil-transmitted helminths (STH) and schistosomiasis (SCH) (8, 9). These large-scale preventive chemotherapy programmes have contributed significantly to reductions in infection intensity, morbidity, and overall disease burden across many endemic countries in the region. However, despite these gains, STH and SCH remain major public health concerns due to continued exposure to transmission sources, including contaminated water, inadequate sanitation, and poor hygiene conditions (10).

Recent estimates indicate that more than 300 million school-age children in sub-Saharan Africa remain at risk of STH infections, while schistosomiasis transmission persists in over 40 African countries, accounting for nearly 90% of global schistosomiasis cases (3, 10). Although MDA programmes have traditionally focused on school-aged children, ongoing transmission occurs because adults, out-of-school children, and other untreated populations continue to act as reservoirs of infection, facilitating reinfection within communities (11, 12).

Kenya has historically experienced a high burden of both STH and SCH. Epidemiological studies show that endemicity remains pronounced in western Kenya, where ecological conditions, poverty, and inadequate sanitation infrastructure favor continued transmission (13, 14). Although Kenya's national school-based deworming programme has achieved significant reductions in infection intensity among school-aged children, recent mapping studies indicate that localized transmission hotspots persist in several counties, including Bungoma, Kakamega, Vihiga, and Trans-Nzoia (13, 15).

The WHO NTD Roadmap 2021–2030 “Ending the neglect to attain the Sustainable Development Goals,” emphasizes integration of NTD interventions into PHC and Universal Health Coverage (UHC) frameworks as a pathway toward sustainability and health system strengthening (2). In Kenya, these global policy shifts coincide with significant health system reforms aimed at strengthening PHC as the foundation of UHC. The Primary Health Care Act (2023), the Kenya Community Health Policy (2020–2030), and the establishment of the Social Health Authority (SHA) under the Social Health Insurance Act (2023) collectively provide an institutional framework for embedding essential health interventions into routine service delivery. These reforms are closely aligned with the Government of Kenya's Bottom-Up Economic Transformation Agenda (BETA), which prioritizes investments in primary health care, preventive services, and community health systems as mechanisms for improving population health and economic productivity (2, 16–19).

Integrating neglected tropical disease (NTD) services into primary health care (PHC) platforms contributes directly to the aspirations of the African Union's Agenda 2063, which envisions “*an integrated, prosperous and peaceful Africa, driven by its own citizens and representing a dynamic force in the international arena”* (20). Specifically, Agenda 2063 emphasizes the importance of healthy, well-nourished citizens with long life expectancy, supported by accessible and quality health systems as a foundation for inclusive growth and sustainable development. Embedding NTD prevention, diagnosis, and treatment within PHC strengthens community-based health systems, expands equitable access to essential health services, and reduces the burden of diseases that disproportionately affect vulnerable populations (2, 21–24). In doing so, such integration advances Aspiration 1 of Agenda 2063, which calls for improved quality of life and well-being through robust social sectors, including health (20).

Despite strong policy support, empirical evidence on the operational feasibility of integrating STH and SCH treatment into PHC systems remains limited in high-burden settings such as Western Kenya (25, 26). Previous research has primarily focused on epidemiological outcomes of deworming campaigns rather than health system readiness, implementation feasibility, cost, effectiveness and operational sustainability of integrated delivery models (26–29). As a result, policymakers lack sufficient context-specific evidence on cost-effectiveness of the two interventions to guide the transition from vertical MDA campaigns to routine PHC-based delivery (25, 28, 29).

Recent studies have begun to address this gap. For instance, Kihara et al. (26) demonstrated that expanding treatment beyond school-based platforms to include community-wide delivery significantly reduced infection prevalence and intensity in Kenya. Similarly, evidence on integrated delivery approach emphasized the importance of integrating deworming interventions with PHC systems to reach underserved populations and sustain gains in transmission reduction (25, 28). Implementation research from Uganda and Tanzania further indicates that facility-based treatment combined with community health worker outreach can improve coverage among adults and marginalized populations often missed by school campaigns (28).

In Kenya, economic evaluations of deworming programs have historically focused on school-based interventions (26, 27). While these programs have demonstrated substantial health benefits and favorable delivery cost, evidence on the cost and effectiveness of integrating deworming into PHC systems remains scarce (26–28). Against this background, this study sought to evaluate the cost and effectiveness of implementing STH and SCH treatment within a PHC-integrated model in Western Kenya. The objective specifically aims to examine whether integrating preventive chemotherapy into routine PHC services is operationally cost effective within existing health system structures, including community health units, primary health facilities, and county health management systems. The study sought to answer the research question, “Is it cost effective to implement STH and SCH treatment through a PHC-integrated delivery model in Western Kenya?”

This study evaluated programme implementation costs and delivery efficiency rather than age-specific epidemiological effectiveness. Age-related differences in infection burden, treatment eligibility, care-seeking behavior and operational feasibility were investigated under separate objectives of the broader mixed-methods evaluation. Since the economic evaluation compared alternative program implementation approaches rather than age-targeted treatment strategies, age-stratified transition probabilities were beyond the predefined analytical scope. The study recommends future economic evaluations incorporating age-specific transition probabilities and sensitivity analyses. The study further recommends future modelling incorporating recurrent financing under the Social Health Authority (SHA), supply chain disruptions, alternative procurement scenarios and sensitivity analyses reflecting interrupted treatment cycles.

## Study objective

To evaluate the cost-effectiveness of integrating soil-transmitted helminths (STH) and schistosomiasis (SCH) treatment into routine primary healthcare (PHC) services compared with campaign-based mass drug administration (MDA) approaches in western Kenya.

## Conceptual framework

The conceptual framework guiding the economic evaluation, as presented in Figure 1 below, was drawn from health systems strengthening and implementation science frameworks, emphasizing the interaction between system inputs, service delivery processes, and implementation outcomes (2, 21, 30). Key inputs include trained health workers, availability of deworming medicines, functional supply chains, digital reporting platforms such as eCHIS, and financing through donor support and normal health facility operational budget (2, 17, 21, 31). These inputs influence service delivery processes such as routine facility-based treatment, community outreach by Community Health Promoters (CHPs), mass drug administration by community drug distributors, routine administration of deworming by health care providers during outpatient and anti-natal care visits and integration of deworming data into health information systems (17, 21, 22).

**Figure 1:**
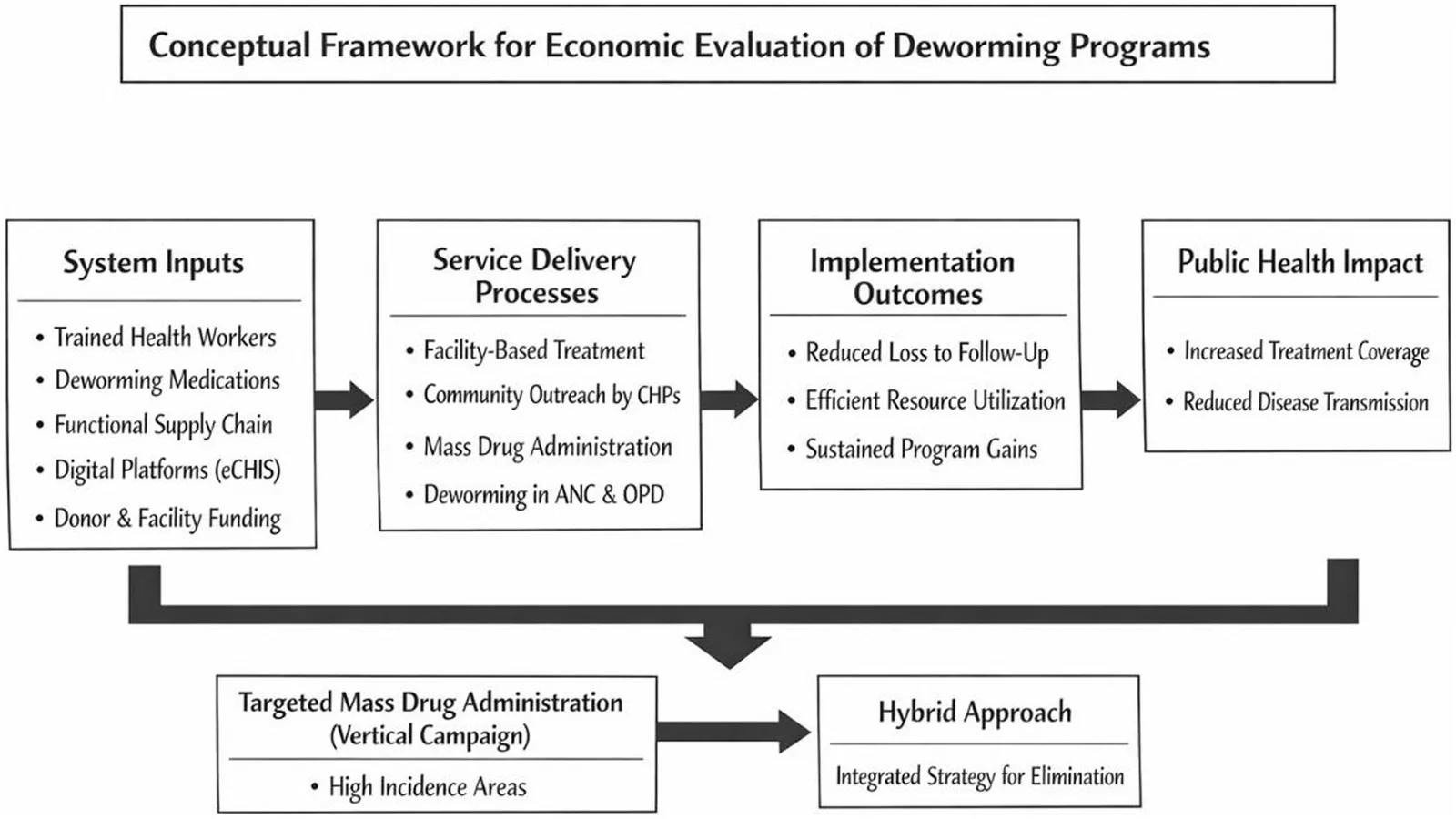
Conceptual framework.

The framework hypothesizes that effective integration of these inputs into PHC workflows leads to improved implementation outcomes such as management of loss to follow-up, effective utilization of the already limited health system costs and maintaining the gains already achieved from previous mass campaign approaches (2, 21). Targeted mass drug administration (Vertical campaign) in wards or sub-counties with high incidences will contribute to broader public health impacts such as sustained treatment coverage and reduced disease transmission. A hybrid of both interventions will contribute to progress toward elimination targets.

## Theory of change

The theory of change guiding the economic evaluation, as presented in Figure 2 below, posits that when STH/schistosomiasis (SCH) treatment is embedded within PHC systems, health-system investment requirements can be reduced while maintaining treatment coverage above WHO-recommended levels, thereby strengthening system sustainability and resilience to shocks, including reduced funding. By leveraging existing PHC infrastructure, trained health personnel, and community health networks, integrated delivery models can reach populations often missed during periodic campaigns, including adults, out-of-school children, and marginalized groups.

**Figure 2:**
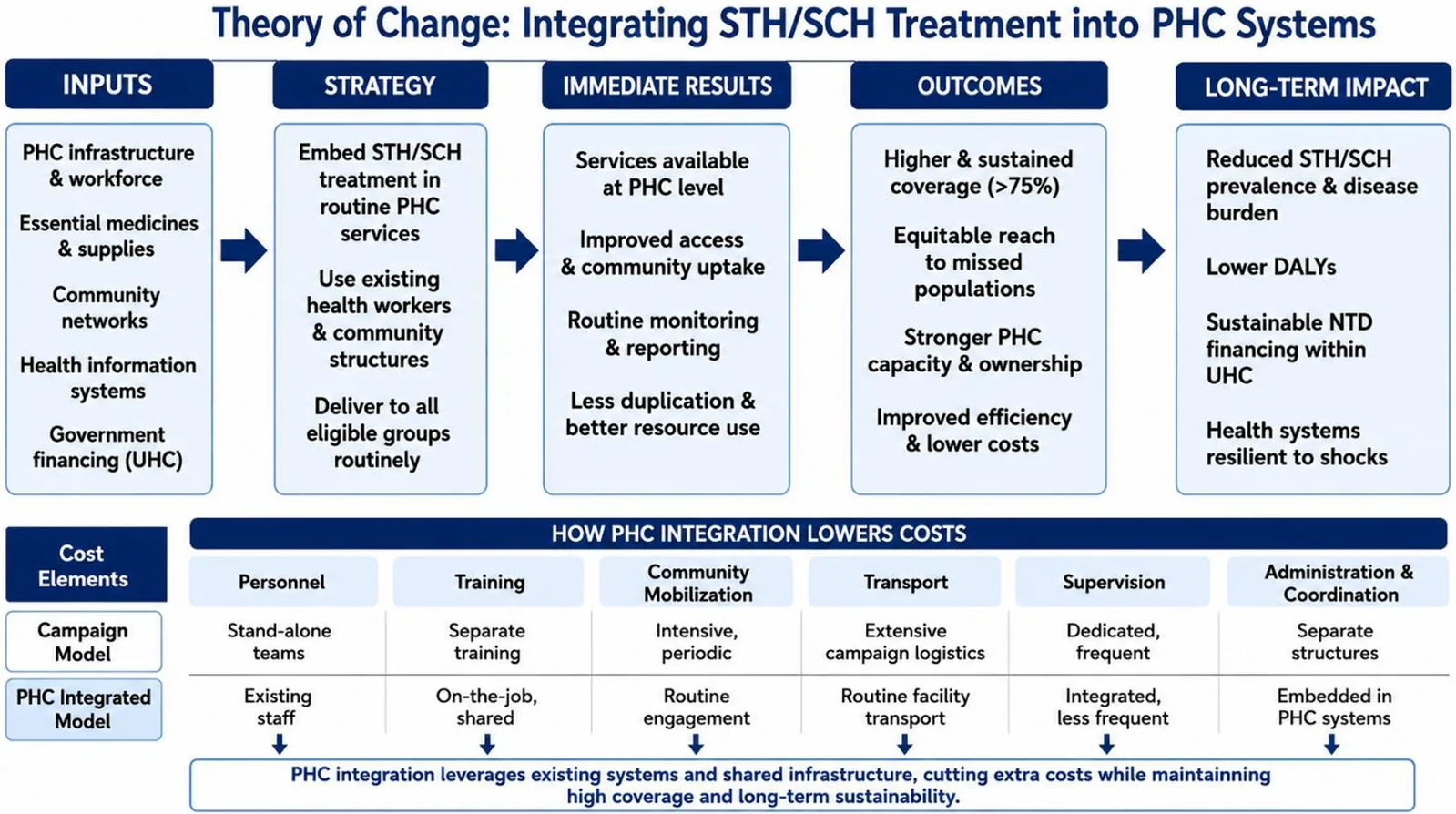
Theory of change integrating STH/SCH treatment into PHC systems.

In the short term, integration is expected to improve service availability, community engagement, and health system ownership of NTD interventions. Over the medium term, these improvements are anticipated to increase treatment coverage and strengthen routine monitoring systems. Overtime, these changes will contribute to long-term impacts including reduced disease prevalence, lower DALYs attributable to helminth infections, and sustainable financing of NTD programs within Kenya's UHC framework.

## Materials and methods

### Planning and designing of the health economic analysis

The health economic analysis plan was developed alongside the quasi-experimental controlled before-and-after (CBA) study design to assess for economic efficiency of integrating schistosomiasis (SCH) and soil-transmitted helminths (STH) treatment into primary health care (PHC) systems compared with the campaign-based mass drug administration (MDA) approach. The analytical framework was guided by the World Health Organization WHO-CHOICE (Choosing Interventions that are Cost-Effective) methodology to ensure internationally comparable cost-effectiveness estimates (32, 33). In addition, the economic evaluation and reporting were aligned with the Consolidated Health Economic Evaluation Reporting Standards (CHEERS) 2022 checklist to strengthen transparency, completeness, and methodological rigor in reporting economic evaluation findings (34, 35). Planning involved defining the analytic perspective, identifying categories relevant to health service delivery, specifying outcome measures such as treatment coverage and disability-adjusted life years (DALYs) averted, and determining the appropriate time horizon for evaluation. Primary cost data were collected from programme implementation records, training activities, logistics operations, and health workforce inputs across the four study counties of Western Kenya (Vihiga, Kakamega, Bungoma, and Trans Nzoia). Given the recurrent nature of SCH and STH infections, a Markov decision-analytic model was incorporated to simulate long-term epidemiological and economic outcomes over a five-year horizon for a cohort of 5,000 individuals. This modelling approach enabled estimation of incremental cost-effectiveness between the PHC-integrated and campaign-based delivery models while accounting for re-infection dynamics, treatment cycles, and long-term public health impact (15, 36).

### Study population

The study included all individuals eligible for schistosomiasis (SCH) and soil-transmitted helminth (STH) treatment in selected wards of western Kenya. Eligible groups comprised preschool-aged children (1–4 years) for STH treatment; school-aged children (5–14 years) for both SCH and STH treatment; and adults (≥15 years) for SCH treatment. Across the 24 evaluation wards, a total target population of 814,059 individuals were targeted and 736,019 treated. Treatment coverage varied by delivery model, reaching 102.3% in campaign-based delivery and 77.3% in PHC-integrated delivery based on administrative reporting.

### Study setting and location

The study covered four counties in Western Kenya: Vihiga, Kakamega, Bungoma, and Trans Nzoia. A total of 24 wards were included with 12 implementing campaign-based mass drug administration (MDA) and 12-wards implementing PHC-integrated treatment. These wards represent a scale subset of the broader regional programme while maintaining analytical comparability between delivery approaches.

### Study comparators

The economic evaluation compared two alternative delivery strategies for SCH and STH treatment. The PHC-integrated delivery (intervention arm) where treatment was delivered through routine primary healthcare facilities and community health services. A total of 872 Community Health Promoters (CHPs), 49 Community Health Assistants (CHAs with 35 trained prior to implementation), 38 health facility in-charges (HFIs), 18 sub-county health representatives, 4 county-level representatives, 3 facilitators and 8 national MOH staff provided treatment during regular service interactions without additional allowances. Recorded data were captured through the routine electronic Community Health Information System (eCHIS) and Kenya Health Information System (KHIS).

The Campaign-based mass drug administration (control arm) treatment was delivered through a vertical MDA campaign lasting five days. A total of 856 Community Drug Distributors (CDDs) conducted household visits and were supported by 44 supervisors and temporary logistical resources. CDDs received campaign allowances. The two models differ primarily in workforce organization, intensity of resource use [USD 133,814 (Kes 17.26 million) for campaign compared to USD 75,519 (Kes 9.91 million) for PHC integration], and delivery duration (3 months PHC integration and 5 days campaign).

### Study perspective

This study adopted a health care system costing perspective, focusing on costs relevant to service delivery and continuity. Health sector costs included organization and delivery of SCH/STH treatment through PHC facilities, training of CHPs and health care workers, health workforce time, programme administration and supervision, recording through eCHIS and DHIS2, and management of adverse events (30, 32, 37). Health outcomes included changes in treatment coverage, service continuity, and effectiveness of PHC-based delivery compared with campaign-based MDA (34). The analysis excluded patient and family costs (out-of-pocket expenses, transport, opportunity costs, caregiver time, productivity losses) and societal/intersectoral perspectives (productivity gains, cross-sectoral savings in education or social protection, long-term human capital development, willingness-to-pay measures) (34, 36, 38). While appropriate for informing implementation decisions, this exclusion represents a limitation. Future evaluations should incorporate these perspectives to capture the full economic and social value of integrated delivery.

### Measurement of outcomes

Health outcomes were estimated using a Markov state-transition model simulating a cohort of 5,000 individuals over five years for SCH and STH infection dynamics. Individuals transitioned annually between infection states as infected, treated or uninfected (37, 39). Given that SCH and STH reinfection occurs frequently in endemic areas, a Markov framework was chosen to capture repeated infection and treatment cycles over time. Working with transition probabilities derived from treatment coverage estimates and baseline infection prevalence, the evaluation enabled quantification of long-term epidemiological gains, DALYs averted, and incremental cost-effectiveness across both delivery strategies (22, 40, 41).

### Methodological limitations

Cost incurred by the patient and family was not factored. Excluding these costs may underestimate the full economic burden or benefits especially if PHC-based delivery reduces travel time, missed work or school days, and reliance on campaign schedules. By not accounting for cross-sectoral spillovers, the analysis likely underestimates the total economic value of integrating SCH/STH treatment into PHC.

The PHC model was assessed using data from a 3-month pilot, while the MDA campaign was evaluated over its standard 5-day cycle. A 5-year analytic horizon was applied to capture medium-term health and economic effects relevant for policy planning and alignment with the Kenya National NTD Plan. This longer horizon allows PHC models with higher upfront investments (training, supervision, system strengthening) to demonstrate value as health benefits accrue gradually over multiple years. This approach is consistent with established health economic evaluation methods and prior schistosomiasis modelling studies (34, 42–46). Pilot data were harmonized using coverage-adjusted annualization to enable like-for-like comparison across the 5-year period. Annualization was necessary to permit direct comparison between delivery strategies operating at different frequencies. Additionally, both delivery models incorporated equivalent implementation cost categories, including personnel, training, supervision, transportation, logistics, administration, monitoring, and commodity distribution. A one-way sensitivity analyses of varied salaries, training, transport, logistics and alternative overhead allocation methods by ±20% was conducted to evaluate uncertainty.

The unit of analysis was cost per person treated per year, rather than cost per treatment delivered. The two treatments were used interchangeably during implementation, and no locally recognized nomenclature existed to distinguish individual treatment events at community level, preventing accurate disaggregation across delivery models. This reflects operational reality and ensures internal consistency for direct comparison between models. An addendum is recommended to explore cost per treatment delivered should more granular data become available in future programmes.

### Costing parameters

The fundamental differences between a five (5) days intensive campaign and a 3-month integrated routine system necessitated distinct costing approaches for each model component. Personnel costs varied markedly. In the campaign model, Community Drug Distributors (CDDs) received allowances during the 5-day campaign period. In contrast, the PHC-integrated model relied on Community Health Promoters (CHPs) and health facility staff who received regular salaries without additional allowances, as treatment delivery was incorporated into routine healthcare services over three months. Healthcare facility staff time allocation also differed with campaign models requiring concentrated effort over short periods while routine models distributed the workload across regular service delivery schedules.

Training requirements reflect these differences. In this study, CDDs were trained 1–2 days before the campaign including preparation for high-volume service delivery within a limited timeframe. Conversely, the routine PHC model emphasized ongoing capacity building embedded within standard professional development programmes, supporting integration of deworming into routine clinical workflows and sustained service quality.

Operational costs also differed substantially. Campaign delivery incurred concentrated expenditures within the five-day period, including intensive supervision, rapid reporting mechanisms, and large-scale drug distribution logistics. The routine model distributed operational costs across three months by embedding deworming into existing service delivery processes, routine patient consultations, and facility-based documentation systems.

Transportation and logistics differ in intensity. Campaigns required rapid drug distribution, frequent supervisory visits, and time-sensitive coordination, whereas the routine model relied on existing supply chains and transport systems within PHC services.

Administrative costs in the campaign model were concentrated during preparation and implementation, while the routine model integrated administrative tasks into existing health system management structures over the three-month period. Figure 3 illustrates the full range of activities and cost components across both delivery models.

**Figure 3:**
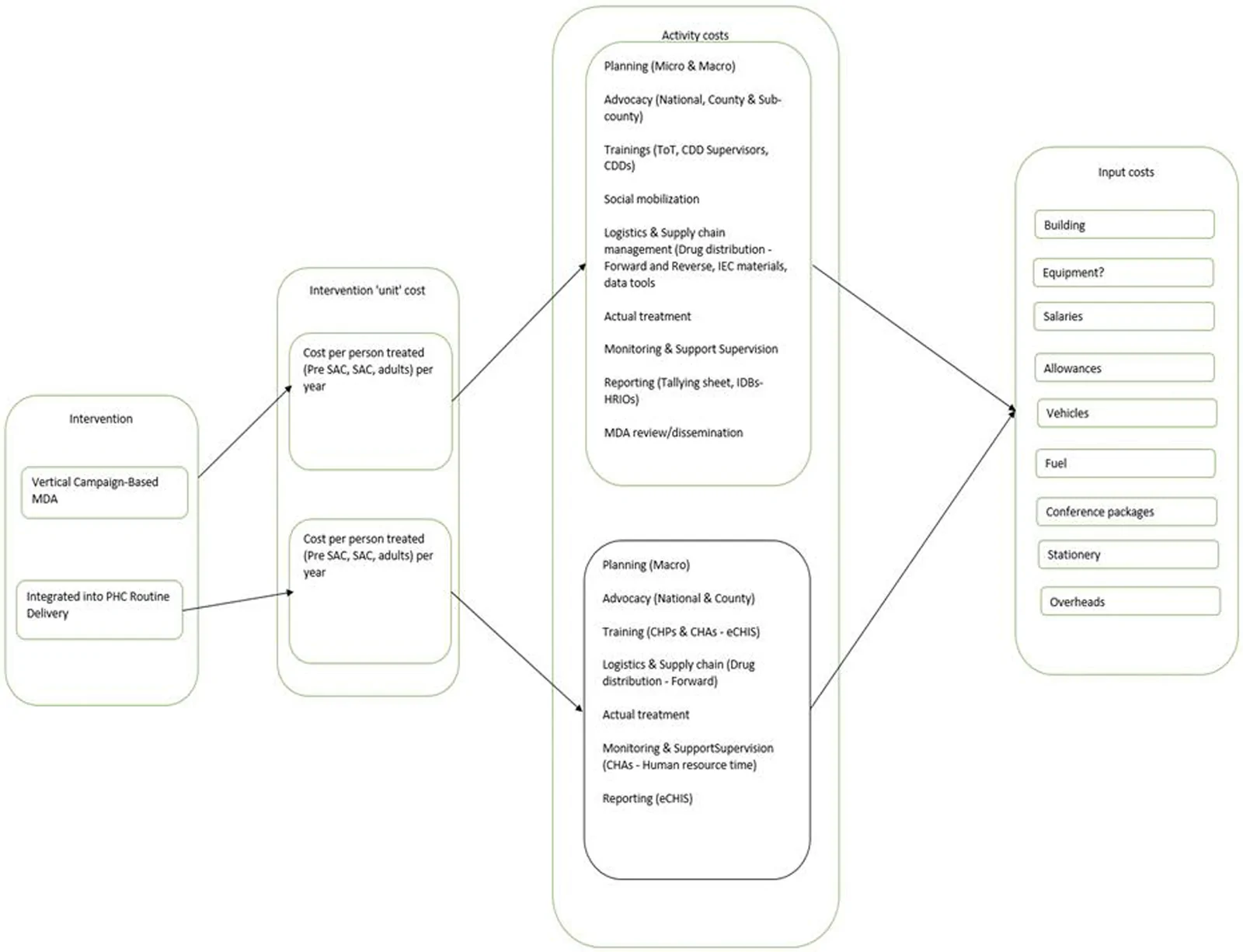
Costing typology.

### Cost analysis approach

Standard unit costs for all resource inputs were developed using local market prices, government salary scales, and international reference prices, where necessary. Costing was conducted from a health-system/program perspective, capturing the resources used to deliver both routine and campaign-based deworming activities.

In the PHC-integrated model, treatment was delivered through routine workflows by health facility staff and Community Health Promoters (CHPs) without recruiting additional personnel. Salaries and stipends for these cadres were therefore classified as routine primary healthcare costs. To account for the small incremental workload associated with deworming (approximately 2–5 min per client for screening, dispensing, counselling, and documentation), a nominal 10% allocation of staff salaries and stipends was initially applied. Although not derived from formal time–motion studies, this estimate is consistent with integrated preventive service delivery (34, 42–46). This allocation was subsequently offset by the 10% tax exclusions normally applied to salaries and stipends, resulting in no additional net labor cost in the PHC model. This approach is consistent with evidence that preventive chemotherapy integrated into routine PHC requires minimal additional staff effort (40, 41, 44).

Qualitative findings did not identify evidence of burnout or deterioration in service quality during PHC integrated implementation. Participants reported that workload was distributed across existing PHC platforms rather than concentrated within individual providers. Nevertheless, Community Health Promoters highlighted increasing reporting responsibilities and supervision demands, indicating that workforce implications warrant continued monitoring. The study recommends future time-motion studies and provider satisfaction assessments to validate these assumptions during national scale-up.

In contrast, the campaign-based model employed Community Drug Distributors (CDDs) who received allowances for the five-day campaign. These allowances, together with supervisory and mobilization activities, were treated as full implementation costs. Shared programme costs were proportionally allocated based on service utilization (5-day campaign period against 4 months PHC integrated pilot period). All costs were converted to 2025 USD (1 USD = 129.3 KES) and adjusted for inflation using Kenya's Consumer Price Index (3.6%–4.5%).

### Sensitivity analysis for costing

Univariate sensitivity analyses were conducted to systematically test the impact of variations in key cost drivers and assess the robustness of cost estimates. Drug procurement costs were analyzed with the understanding that medication prices were likely similar across both delivery models, with variations of ±20% applied uniformly to capture potential market fluctuations. Healthcare worker salaries were tested with a ±20% variation to account for possible changes in compensation levels or regional differences in salary scales. Training costs were also subjected to a ±20% variation to reflect potential variability in training approaches, duration, and resource requirements. Transportation and logistics costs were varied by ±20% to represent differences in geographic accessibility and fuel price volatility.

The analysis compared results generated using allocation criteria based on service volume, staff time utilization, physical space occupied, and patient load to determine the most appropriate and realistic approach. These sensitivity tests provided insights into how variations in cost allocation methodologies influenced the overall comparative cost estimates between the PHC-integrated and campaign-based delivery models.

### Costing assumptions and limitations

A key modelling assumption was that differences in delivery intensity and duration between the 5-day campaign-based MDA and the 3-month PHC-integrated delivery could be standardized through annualization within the Markov modelling framework. Coverage and treatment effects were expressed as time-adjusted transition probabilities, allowing both delivery platforms to be compared over a common analytic period despite differing implementation timelines. Staff productivity was assumed to remain constant during the study period, although actual performance may vary due to factors such as experience, seasonal workload changes, or staff motivation. Similarly, facility utilization patterns were assumed to reflect typical operational conditions, though these may fluctuate depending on community acceptance, service demand, or competing health priorities.

Additional limitations were acknowledged in the costing approach. Potential economies of scale in the routine PHC model such as efficiencies gained by delivering deworming alongside services like vaccination, nutrition screening, or child health consultations, may not have been fully captured in the cost-per-person estimates. Exchange rates were assumed to remain stable during the analysis period, although currency fluctuations could affect cost comparability. Some cost components may also have been underestimated, including informal or opportunity costs borne by communities, patients, and health workers, particularly volunteer contributions. Seasonal variations in programme intensity and uncertainties in allocating shared infrastructure or capital costs may also have influenced cost estimates, as allocation of shared resources inevitably involves some level of judgment.

### Markov transition probability model

The cost-effectiveness analysis focused on programmatic outcomes based on the total resources used to deliver treatment for soil-transmitted helminths (STH) and schistosomiasis (SCH). A Markov transition probability model was used to project long-term cost-effectiveness. Because the two delivery strategies operated over different implementation periods, costs and treatment coverage were annualized to produce comparable inputs for modelling. For the PHC-integrated model, data collected during the 3-month pilot period were extrapolated to annual estimates based on expected routine service delivery across a 12-month cycle, assuming the pilot represented steady-state PHC operations after initial setup. For the campaign-based model, costs and output from the 5-day MDA campaign were annualized, assuming one campaign per year, consistent with national practice. Non-recurrent start-up costs such as training and system adjustments were distributed across years to avoid inflating annual PHC costs.

The annualization process produced harmonized estimates of cost per person treated and treatment coverage, which served as yearly transition inputs in the Markov model. Individuals moved between health states such as infected, treated, uninfected, and reinfected, on an annual cycle (41), reflecting the recurrent nature of helminth infections. This modelling approach follows established methods used in STH programme evaluation (47), which simulate changes in prevalence and morbidity over multi-year horizons. As illustrated in Figure 4 below, these models demonstrate the progression of helminth-related morbidity in the absence of preventive chemotherapy and the potential effects of preventive chemotherapy under varying infection-intensity and exposure scenarios. Within this framework, health benefits were estimated through reductions in morbidity risk rather than directly measuring infections averted, as longitudinal infection data were not available.

**Figure 4:**
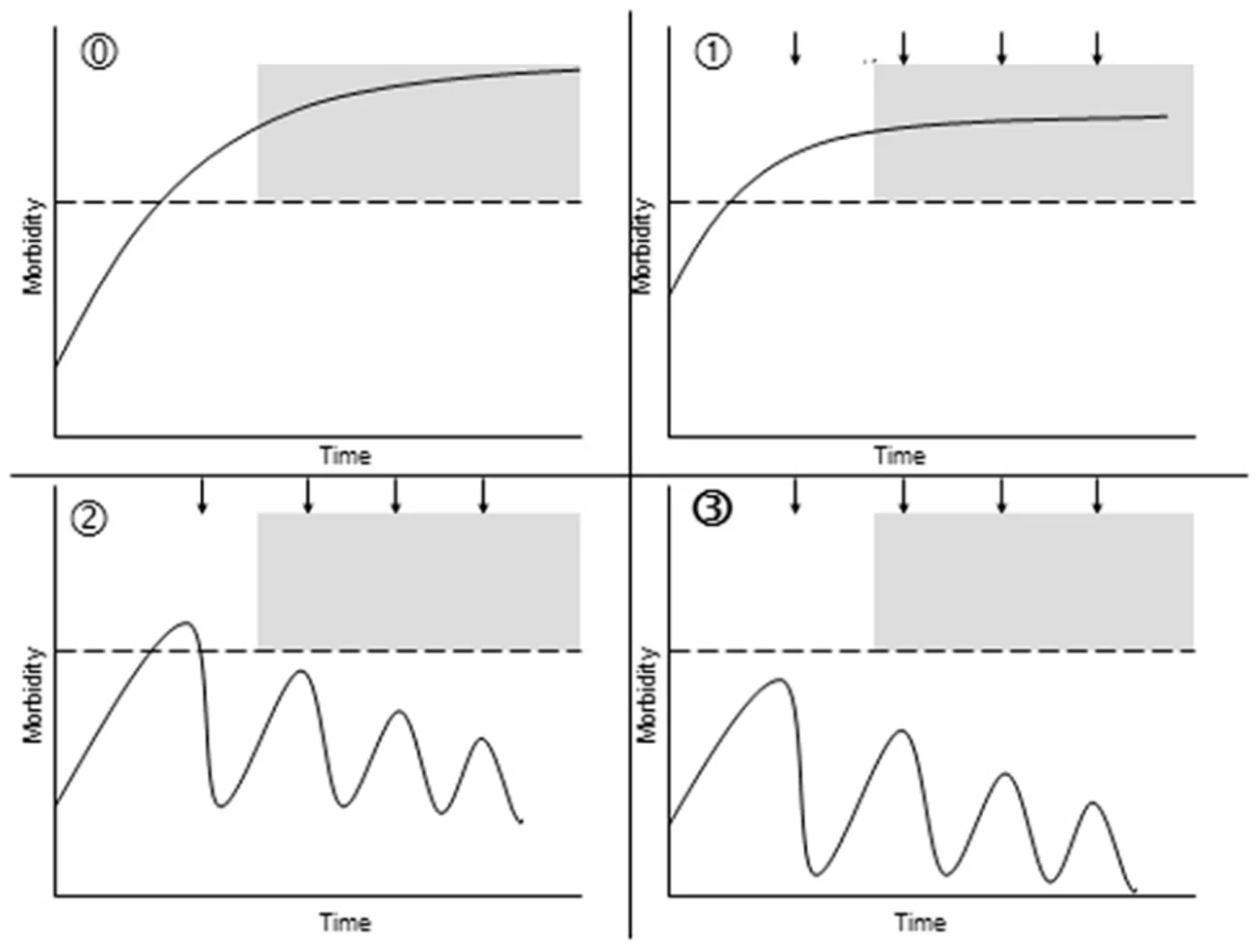
Adopted from montresor et al. (47), illustration of helminth-related morbidity progression without preventive chemotherapy (0) and the effects of preventive chemotherapy under varying infection intensity and exposure scenarios (1–3).

### Transition probability estimation

The Markov model applied annual cycles over a five-year horizon to project changes in schistosomiasis (SCH) and soil-transmitted helminth (STH) prevalence using one-year transition probabilities, consistent with Montresor et al. (47). Baseline prevalence estimates were informed by recent precision mapping in western Kenya involving more than 46,000 school-aged children across Kakamega, Bungoma, Vihiga, and Trans Nzoia counties (13). The survey reported relatively low overall prevalence (3.2% for SCH and 7.6% for any STH), mostly light-intensity infections, but with significant spatial heterogeneity and localized hotspots exceeding 60%, providing realistic prevalence parameters for low-prevalence post-control settings with residual transmission (13).

Transition probabilities between infected and treated or uninfected states were estimated using a coverage-adjusted approach, reflecting that differences between intervention and control arms were driven primarily by treatment coverage and delivery strategy rather than drug efficacy. Annual treatment coverage for the campaign-based model was derived directly from the single MDA round and treated as yearly coverage, while coverage for the PHC-integrated model was extrapolated from the three-month observation period using a utilization factor to account for seasonal variation in health facility attendance.

### Cost-Effectiveness analysis, and ICER estimation

The primary effectiveness outcome for this economic evaluation was disability-adjusted life years (DALYs) averted, which formed the basis for estimating the incremental cost-effectiveness ratio (ICER) and informing recommendations. DALYs were selected because they capture the broader health impact of controlling schistosomiasis (SCH) and soil-transmitted helminths (STH) by translating reductions in infection prevalence and intensity into gains in healthy life years. This measure also enables comparison with other health interventions and supports resource allocation decisions within the health sector (48–50). An ingredient-based costing approach was used to estimate the unit cost of each delivery strategy by systematically identifying and valuing all resources required for treatment delivery. Total and incremental costs were calculated over a five-year analytic horizon and discounted at 3% annually, consistent with international economic evaluation practice (51). Differences in implementation timelines were harmonized by annualizing costs and outputs from the three-month pilot using coverage-adjusted projections.

DALYs were estimated using standard burden-of-disease methods, incorporating years lived with disability (YLDs) and, where applicable, years of life lost (YLLs) (48, 49). Current WHO methods draw on Global Burden of Disease (GBD) estimates for YLDs, with YLDs calculated from disease prevalence and corresponding disability weights without age weighting or discounting (52). Disability weights were therefore derived from GBD 2019 framework, with no age-weighting applied (52). Life expectancy values were obtained from standard WHO life tables, while disease duration was derived from modelled infection states rather than direct clinical observation due to the absence of longitudinal parasitological data during the pilot (53).

The Markov model assumed that transitions depended on the current infection state, with transition probabilities remaining constant but tested through sensitivity analyses. Differences in effectiveness between delivery strategies were driven primarily by treatment coverage, assuming comparable drug efficacy (47, 54). The use of modelling to estimate the health effects of preventive chemotherapy is consistent with established economic evaluations of schistosomiasis and STH interventions, including models that estimate DALYs averted under alternative treatment strategies (54–56). The ICER was calculated below as the additional cost required to avert one DALY when comparing the PHC-integrated delivery model with the campaign-based approach.

$$\mathrm{ICER}=\frac{C_{\mathrm{PHC}}-C_{\mathrm{Campaign}}}{DALYs_{\mathrm{PHC}}-DALYs_{\mathrm{Campaign}}}$$

Where C_PHC_ and C_Campaign_ represent the total discounted costs of the PHC-integrated and campaign-based models, respectively, and DALYs_PHC_ and DALYs_Campaign_ represent the total disability-adjusted life years (DALYs) under each strategy. Both costs and DALYs were discounted at 3% annually, consistent with commonly applied economic evaluation practice (51). A positive ICER indicates the additional cost per DALY averted by the PHC-integrated model relative to the campaign approach.

Cost-effectiveness was evaluated against a Kenya-relevant opportunity-cost perspective rather than relying entirely on conventional GDP-based thresholds. Econometric estimates place this threshold at about US$908 per DALY averted, while some Kenyan analyses apply approximately 0.5 × GDP per capita (about US$919) (51, 57). Interventions with ICERs below this range were considered cost-effective. Sensitivity analyses varied key cost and effectiveness parameters by ±20%, tested discount rates of 0%, 3%, and 6%, and conducted threshold analyses to determine when the preferred strategy would change (55).

### Data sources for costing and cost-effectiveness

Standardized cost collection forms were developed to capture detailed information on resource utilization, staff time allocation, and operational expenses across all identified cost centers. Secondary data sources provided supplementary cost information through administrative records, financial reports, procurement databases, and existing program documentation. Government salary scales, standard drug prices from official procurement channels, and official exchange rates were utilized for cost standardization across different facilities and time periods. Historical program data informed estimates for activities that were not directly observable during the study period. Given that deworming was integrated into routine primary healthcare (PHC) services, no time-motion study was conducted. Instead, workforce costs were based on the minimal staff input required for administering the deworming medication, providing routine prescriptions where necessary, and recording the intervention as part of standard PHC service delivery.

### Sample size calculation

The cost-effectiveness analysis for this study was restricted to the 24 evaluation wards (12 PHC integrated intervention wards and 12 control wards delivering the vertical campaign-based model). A hypothetical cohort of 1,000 individuals per year was used as the analytic denominator for the Markov simulation, consistent with global economic evaluation best practices. Over the five-year analytic horizon, the Markov model simulated 5,000 individuals.

### Ethical considerations

The evaluation aligned to ethical standards for research involving human participants. Informed consent was obtained from all respondents prior to participation in surveys or interviews. Participation was voluntary, and individuals could withdraw at any time without affecting access to health services. Data was anonymized and treated confidentially. Only authorized research personnel had access to raw data.

### Data quality control and pretesting of tools

Data collection tools were pretested prior to implementation. Digital questionnaires were programmed in KOBO Toolbox with validation checks, skip logic, geotagging, and time stamps to minimize entry errors. Supervisors monitored data submissions daily using real-time dashboards. Qualitative research teams underwent joint training and conducted pilot interviews to standardize data collection procedures. Cross-referencing of records across eCHIS, facility registers, and survey datasets helped identify and remove duplicate treatment records. Daily briefing meetings between the study team were held to review field progress, identify emerging data quality concerns, and agree on corrective actions-thereby safeguarding the overall integrity and reliability of both qualitative and quantitative data throughout the study.

### Data storage and security

All datasets were encrypted and securely stored within institutional servers. Access to identifiable information was restricted to authorized research staff, and analyses were conducted using de-identified datasets to ensure confidentiality.

## Findings and discussions

### Rationale and cost parameters

The PHC-integrated model incurred a total annual cost of KES 9,741,999 (USD 75,519), substantially lower than KES 17,261,764 (USD 133,814) for campaign-based delivery. Costs were consistently higher for campaigns across personnel, training, community mobilization, transport, and supervision due to stand-alone training, intensified mobilization, and extensive transport and supervisory needs. PHC integration benefited from embedded systems and shared infrastructure, lowering incremental costs. Administration and coordination costs were similar across models, with PHC relying on embedded coordination and campaigns on dedicated structures. For sensitivity analysis, all cost components were varied by ±20% to reflect plausible uncertainty in fuel prices, staff availability, allowance rates, and mobilization intensity, ensuring findings were robust to realistic variations. Table 1 below presents the annual cost parameters used to compare PHC-integrated and campaign-based delivery models.

**Table 1:** Annual cost parameters of PHC-integrated and campaign based delivery models.

Parameter	PHC-Integrated (KES)	PHC-Integrated (USD)	Campaign-Based (KES)	Campaign-Based (USD)	Range (±20%)	Notes
Personnel cost per round	1,420,000	11,008	4,860,000	37,674	±20%	Allowances/per diems only for campaigns; ±20% reflects uncertainty in staff numbers and allowance rates
Training cost	820,000	6,357	1,950,000	15,116	±20%	Integrated on-the-job training vs. stand-alone campaign; ±20% captures variation in duration and participants
Community mobilization	610,000	4,728	2,140,000	16,589	±20%	Intensified mobilization for campaigns; ±20% reflects outreach intensity and media cost uncertainty
Transport & logistics	2,330,000	18,062	5,180,000	40,155	±20%	Fuel, supervision travel, drug transport; ±20% reflects fuel price volatility and route coverage
Monitoring & supervision	1,180,000	9,147	1,760,000	13,643	±20%	Higher supervision intensity in campaigns; ±20% reflects frequency of field visits and staffing
Administration & coordination	1,380,999	10,707	1,371,764	10,633	±20%	Embedded PHC coordination vs. dedicated campaign; ±20% reflects overhead and management time
**Total annual cost**	**9,741,999**	**75,519**	**17,261,764**	**133,814**	**–**	**Field-derived totals; not varied directly in sensitivity analysis**

## Cost and coverage performance

This analysis compares total programme costs and average cost per person treated for the vertical campaign-based model and the PHC-integrated MDA model implemented across 24 wards in western Kenya. The campaign model was implemented in 12 wards across Bungoma, Kakamega, Trans Nzoia, and Vihiga, targeting 426,207 people. Through intensive community mobilization within a short delivery window, 436,059 individuals were treated, achieving 102.3% coverage, a common MDA outcome when real-time attendance exceeds planning estimates due to factors such as population movements. The total campaign cost was KES 17,261,764.45, equivalent to KES 39.59 per person treated.

In contrast, the PHC-integrated model, implemented in 12 routine wards, delivered treatment through existing health system platforms such as outpatient departments, maternal and child health clinics, outreach services, and community–facility referral systems. From a target population of 387,852, 299,960 individuals were treated, achieving 77% coverage. The total programme cost was KES 9,741,999.47, corresponding to KES 32.48 per person treated. While campaigns achieved higher coverage, PHC integration reduced costs by leveraging existing staff, infrastructure, and outreach systems. Together, the two approaches treated 736,019 people, illustrating complementary strengths of scale versus cost efficiency. Table 2 below compares the two delivery models in terms of coverage, overall costs, and cost per person treated.

**Table 2:** Coverage, cost, and cost per person treated.

Indicator	PHC-Integrated Model	Campaign-Based Model	Computation/Notes
Target population	387,852	426,207	Programme planning targets
Number treated	299,960	436,059	Verified treatment registers
Coverage (%)	77%	102.30%	Treated ÷ target population
Total programme cost (KES)	9,741,999.47	17,261,764.45	Sum of all MDA-specific inputs
Cost per person treated (KES)	32.48	39.59	Total cost ÷ number treated
Cost perspective	Direct programme cost	Full campaign cost	PHC excludes routine system inputs

Cost per person treated reflects direct financial costs attributable to MDA delivery. For the PHC-integrated model, routine health system resources were leveraged but not monetized, illustrating reduced incremental expenditure compared with the stand-alone campaign approach.

## Total and average costs across delivery models

This analysis compares financial costs across 24 wards (12 per model) implementing two MDA delivery strategies: PHC-integrated routine delivery and vertical campaign-based delivery. The PHC-integrated model captured only incremental MDA costs, as it relied on existing PHC staff, infrastructure, and outreach systems, whereas the campaign model required stand-alone operational inputs such as additional allowances, intensive mobilization, dedicated logistics, and expanded supervision, increasing overall costs.

Across the 12 PHC wards, 299,960 people were treated out of 387,852 targeted (77.3% coverage) at a total cost of KES 9,741,999.47 (USD 75,465.11). This corresponds to an average cost of KES 811,833 per ward (USD 6,295.60) and KES 32.48 (USD 0.25) per person treated. In contrast, the campaign model treated 436,059 people against a target of 426,207 (102.3% coverage) with total expenditure of KES 17,261,764.45 (USD 133,871.55), equivalent to KES 1,438,480 per ward (USD 11,150.62) and KES 39.59 (USD 0.31) per person treated. Overall, PHC integration demonstrated lower costs per administrative unit and beneficiary, while campaigns achieved broader and faster population reach, as summarized in Table 3.

**Table 3:** Total and average cost across delivery models.

Cost Indicator	PHC-Integrated Model (12 wards)	Campaign Model (12 wards)	Key Interpretation
Total programme cost (KES/USD @129)	9741999.47 (USD75465.11)	17261764.45 (USD133871.55)	Campaign delivery required higher operational inputs
Average cost per ward (KES/USD)	811833.29 (USD 6295.6)	1438480.37 (USD 11150.62)	Higher logistics and supervision requirements
Cost per person treated (KES/USD)	32.48 (USD 0.25)	39.59 (USD 0.31)	PHC integration is more cost-efficient
Cost difference (Campaign – PHC)	7,519,764.98 (USD 58,406.44)		Additional resources required for campaign delivery

## Cost-Effectiveness analysis: infections and DALYs averted

Cost-effectiveness was assessed by linking programme costs with estimated health outcomes: infections and DALYs averted, based on observed treatment coverage and epidemiological assumptions. A 5-percentage-point reduction in infection prevalence was assumed for treated individuals (About 5 infections prevented per 100 treated), reflecting moderate but persistent infection levels reported in western Kenya (8% STH and 3% schistosomiasis prevalence) (13). This parameter aligns with programme evidence showing sustained reductions following repeated deworming interventions (13, 14).

Model-specific DALY reductions per treated person were applied to reflect delivery platform differences. The PHC-integrated model (0.049 DALYs/person) assumes greater continuity of care, follow-up, and reduced reinfection risk through routine services, while the campaign model (0.043 DALYs/person) reflects episodic delivery with higher reinfection risk (33, 51, 54). These values are consistent with GBD disability weights and economic evaluations of preventive chemotherapy, where individual DALY losses are small, but population-level gains are substantial at high coverage (52, 55).

Applying these parameters, the PHC-integrated model treated 299,960 individuals, averting 14,998 infections and 14,698 DALYs, while the campaign model treated 436,059 individuals, averting 21,803 infections and 18,751 DALYs. The campaign therefore produced 4,053 additional DALYs averted but at an incremental cost of KES 7,519,764.98, resulting in an ICER of KES 1,855 per DALY (USD 14.38). Although more resource-intensive, the campaign approach remains highly cost-effective relative to commonly cited opportunity-cost thresholds for Kenya, particularly where rapid scale-up and high population reach are required, as demonstrated below in Table 4.

**Table 4:** Comparative cost-effective outcomes.

Indicator	PHC-Integrated Model	Campaign Model	Computation/Assumption
Population targeted	387,852	426,207	Programme targets
Population treated	299,960	436,059	Treatment registers
Treatment coverage	77.34%	102.30%	Treated ÷ targeted
Total programme cost (KES)	9,741,999.47	17,261,764.45	Financial records
Cost per person treated (KES)	32.48	39.59	Total cost ÷ treated
Estimated infections averted	14,998	21,803	Treated × 0.05
DALYs per treated person	0.049	0.043	Model assumption
Total DALYs averted	14,698	18,751	Treated × DALY value
Incremental cost (KES)	–	7,519,764.98	Campaign – PHC
Incremental DALYs	–	4,053	Difference in DALYs
ICER (KES/DALY)	–	1,855	Incremental cost ÷ DALYs
ICER (USD/DALY)	–	14.38	Exchange rate: 129 KES/USD

## Cost-Effectiveness projections based on a Markov framework

### Rationale for the modelling framework

Two parallel hypothetical cohorts of 1,000 individuals were simulated for the PHC-integrated and campaign-based delivery models, representing the programme's 12-ward implementation arms. The model used annual cycles over a 5-year horizon to reflect repeated MDA rounds. Treatment coverage parameters were derived from programme data (77.34% for PHC and 102.3% for ecampaigns, capped at 1.0 for modelling validity). Reinfection rates were assumed constant with ±20% sensitivity variation, and treatment cure probabilities were derived from coverage and drug efficacy assumptions.

Health outcomes were estimated using GBD-aligned disability weights (DW) reflecting infection severity: 0.006 for light infection, 0.024 for moderate infection, and 0.049 for heavy infection, where 0.006 corresponds to the GBD light/mild infection weight (58, 59). A 3% discount rate was applied to both costs and health outcomes (60). Cost-effectiveness results were interpreted against an assumed opportunity-cost threshold of approximately USD 500–700 per DALY averted, informed by empirical approaches to estimating health opportunity costs in low- and middle-income countries (61, 62), with the key Markov model parameters and baseline assumptions presented in Table 5.

**Table 5:** Key Markov model parameters and baseline assumptions.

Model Parameter	Baseline Value	Range/Distribution	Source/Reference	Interpretation
Cycle length & Time horizon	1 year &; 5 years	Fixed	Montresor et al. (47); WHO (40)	Aligns with annual MDA cycles; Captures cumulative effects of repeated treatment
Health states	4 states	Fixed	Montresor et al. (47)	No infection, light, moderate, heavy infection
Treatment coverage – PHC	77.34%	±10%	Programme data	Reflects routine PHC service delivery
Treatment coverage – Campaign	102.3% (capped at 1.0)	±10%	Programme data	Ensures probabilistic validity
Drug efficacy (STH); Reinfection rate	High for hookworm (ABZ); Constant	Scenario tested: ±20%	Okoyo et al. (15); Montresor et al. (47)	Albendazole is more effective than mebendazole; Conservative reinfection assumption
Treatment Cure Probability– PHC	0.7734	Derived	Coverage × efficacy	Reflects routine service uptake
Treatment cure Probability– Campaign	1.0 (max)	Fixed	Assumption	Biological upper bound
Disability weight – light infection	0.006	Fixed	Turner et al. (58); GBD (52, 59)	Mild morbidity burden
Disability weight – moderate infection	0.024	Fixed	Turner et al. (58); GBD (52, 59)	Moderate morbidity
Disability weight – heavy infection	0.049	Fixed	Turner et al. (58); GBD (52, 59)	Severe morbidity
Discount rate	3%	0%–5%	Rushby et al. (60)	Standard economic evaluation practice
DALYs per treated – PHC	0.049	±20%	Turner et al. (58)	Reflects sustained morbidity reduction
DALYs per treated – Campaign	0.043	±20%	Turner et al. (58)	Reflects higher reinfection risk
Cost-effectiveness threshold (Kenya)	USD 500–700 per DALY	Context-specific	Ochalek et al. (61); Lomas et al. (62)	Opportunity-cost benchmark

### Transition probability estimates

Annual transition probabilities governed movement between infection-intensity states, reflecting changes in infection prevalence and intensity over time rather than direct parasitological outcomes such as egg reduction or cure rates. Because drug efficacy is typically reported using parasitological indicators, these measures were translated into probabilities of transitioning to lower-intensity or uninfected states, which are more appropriate for population-level economic evaluation.

Baseline transition structures were adapted from published Markov models of helminth infection dynamics, which estimate annual shifts in infection intensity following preventive chemotherapy (47, 63). The matrices were weighted to represent the dominant helminth species in western Kenya—*Schistosoma mansoni*, hookworms, *Ascaris lumbricoides*, and *Trichuris trichiura* (47). Species-specific drug efficacy estimates for praziquantel, and albendazole were drawn from Kenyan evidence MDA programme performance and used to adjust the probability of transitioning to the “not infected” state after treatment (15, 27). Reinfection dynamics were incorporated through annual probabilities of returning to infected states, reflecting persistent endemic transmission. These reinfection parameters were assumed constant across cycles and informed by Kenyan longitudinal studies, consistent with standard modelling practice when contemporaneous reinfection data are limited (47, 63).

The model population represents community-wide MDA delivery in western Kenya (Bungoma, Kakamega, Trans Nzoia, and Vihiga counties), covering pre-school-aged children (1–4 years), school-aged children (5–14 years), and adults (≥15 years) (13, 64). Baseline epidemiological inputs were derived from precision mapping studies, which estimated ∼7.6% prevalence for soil-transmitted helminths and ∼3.2% for *S. mansoni* among school-aged children (13). Infection intensity distributions used to initialize the Markov cohorts were approximated as 89% not infected, 7% light infection, 3% moderate infection, and <1% heavy infection, based on longitudinal prevalence–intensity patterns in western Kenya (47, 65).

Although more recent mapping indicates declining and increasingly focal transmission in western Kenya (13, 27), baseline prevalence was held constant across model cycles as a conservative assumption. This approach avoids over-attributing reductions in infection to programme interventions while ensuring that coverage-driven changes in infection intensity and morbidity remain the primary drivers of health outcomes in the cost-effectiveness analysis.

### Disability weight estimates

Disability weights applied were: 0.006 for light infection, 0.024 for moderate infection and 0.049 for heavy infection. 0.006 corresponds to the GBD light/mild infection disability weight (52, 58, 59). Values for moderate (0.024) and heavy (0.049) are derived by applying multipliers to the baseline GBD value, reflecting greater morbidity severity in moderate and heavy infection states. These multipliers are consistent with approaches in published burden and modelling literature where intensity correlates with higher disability (66). Mortality was not modelled because the model focused on morbidity-related health gains (66, 67). Figure 5 below illustrates the Markov states and disability weights applied annually.

**Figure 5:**
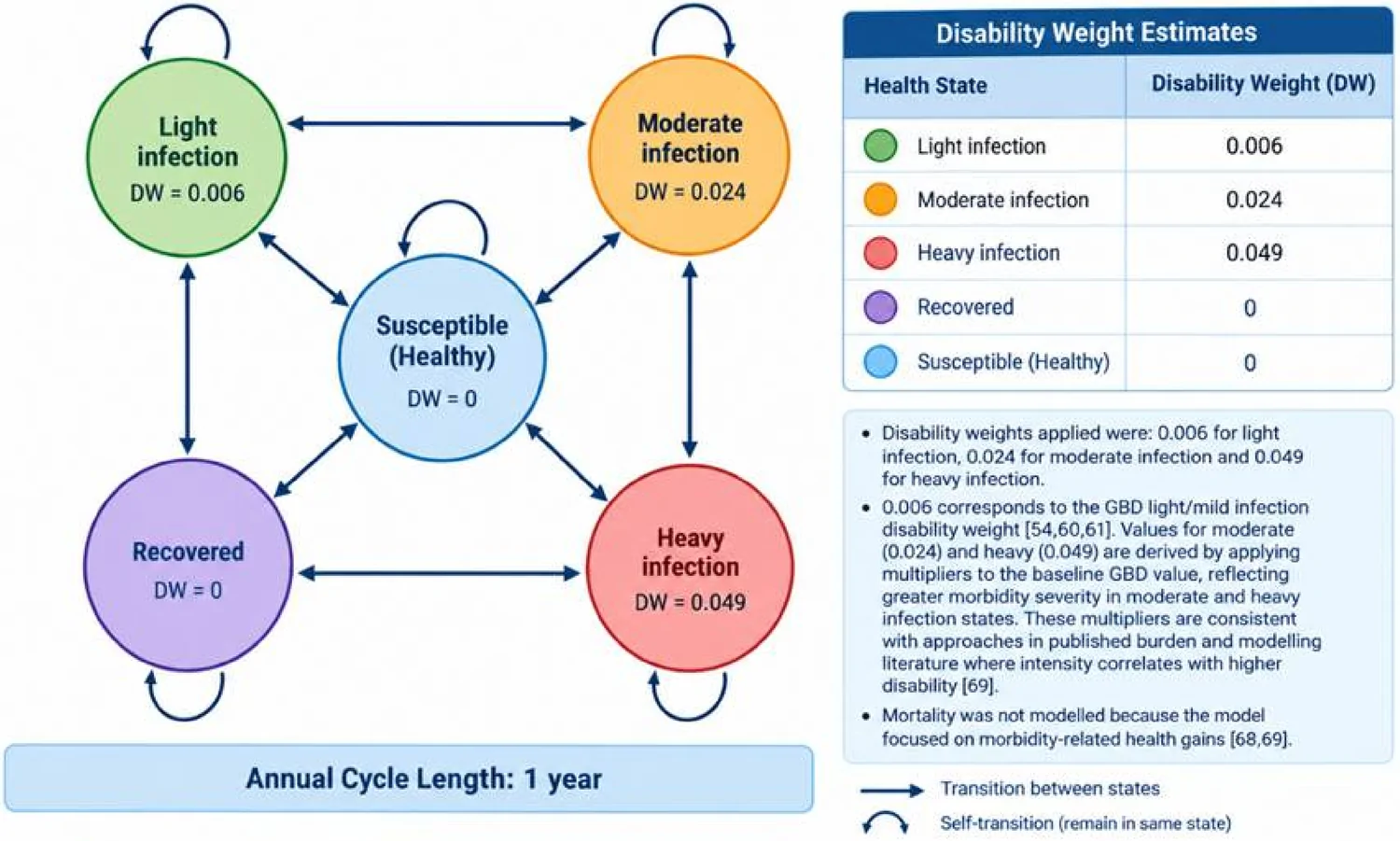
Core Markov state (annual cycle length) and disability weights used in model 2.

### Costing and comparative analysis for the model

Costs were estimated from the programme perspective and disaggregated by activity category. All costs were collected in Kenyan Shillings and converted to USD using the 02 October 2025 OANDA exchange rate (KES 129 per USD). Unit costs were calculated per person treated, stratified by delivery modality but not by age, as drugs are donated and delivery activities are largely age neutral. The incremental cost-effectiveness ratio (ICER) compared PHC-integrated MDA with campaign-based delivery:

$$ICER=\frac{C_{PHC}-C_{Campaign}}{DALYs_{Campaign}-DALYs_{PHC}}$$

ICERs were interpreted against an opportunity-cost-based threshold of USD 1,234 per DALY averted, representing an assumed estimate of Kenya's health system marginal productivity (57, 61).

### Uncertainty and limitations

Uncertainty was examined using deterministic sensitivity analysis (±10% or ±20% variation in key parameters) and probabilistic sensitivity analysis with 1,000 Monte Carlo simulations, which showed that treatment coverage and unit delivery costs were the main drivers of decision uncertainty. A limitation of the model is that some transition probabilities were derived from evidence generated before 2016 and adjusted using more recent Kenyan data rather than being based entirely on contemporary longitudinal observations from the study wards. Additionally, although national prevalence has generally declined since 2016, spatial heterogeneity has increased, and the model does not explicitly capture micro-spatial transmission hotspots or behavioral differences. As a result, the findings represent average programme-level effects useful for strategic and economic decision-making rather than precise predictions at the local level.

### Key assumptions

Several assumptions underpinned the analysis. Albendazole and mebendazole were treated as interchangeable for STH treatment, consistent with WHO guidance, although albendazole shows higher efficacy against hookworm, which has a low prevalence in western Kenya. Drug efficacy was assumed uniform across individuals regardless of prior treatment history, and reinfection rates were held constant due to limited age-specific longitudinal data. Differences in mortality across infection-intensity categories were not modelled because case-fatality rates for STH and SCH are negligible. Deworming was considered a routine nursing responsibility, so no additional labor costs were included beyond a 10% adjustment offset by payroll deductions. Finally, the PHC delivery model was assumed to use existing facility resources, implying no incremental overhead costs for infrastructure, utilities, or additional staff recruitment.

### 5-Year cohort simulation

In the Markov simulation, each delivery arm initially included 1,000 individuals (scaled to 5,000 for interpretation) who transitioned annually among four infection states using adjusted matrices incorporating treatment coverage and efficacy. Under the campaign delivery model (coverage 102.3%), approximately 5,115 individuals were treated per 5,000 simulated, reflecting the common MDA phenomenon where coverage exceeds 100% due to population mobility and denominator underestimation. Over five years, the proportion of individuals in the “not infected” state increased rapidly, accompanied by substantial declines in moderate and heavy infections, resulting in greater population-level reductions in infection intensity and higher DALYs averted.

In contrast, the PHC-integrated model (coverage 77.3%) treated 3,867 individuals annually out of 5,000, producing more gradual shifts toward the “not infected” state due to lower coverage, although treated individuals still experienced strong per-person health gains owing to high drug efficacy: repeated treatment rounds progressively reduced infection severity, making heavy infections rare by year five. Overall, annual preventive chemotherapy in both arms shifted infection distributions toward lighter intensity states, with heavy infections becoming uncommon and moderate infections declining substantially by the fifth year; however, while the campaign model achieved faster population-level gains, PHC integration produced strong individual-level benefits but slower aggregate impact due to more limited reach.

### Transition probability matrix

To ensure that the Markov model accurately represents the real-world performance of the two delivery modalities assessed in this study were calibrated using empirically observed treatment coverage and the resulting effective probability of clearing infection after treatment.

For the PHC-integrated model, the mean treatment coverage was 77.34%, producing an effective probability of 0.7734 for infection cure following treatment. This probability applies only to people who are already infected (light, moderate, or high intensity). It determines how much of each infected group transitions into the “Not infected” state during each model cycle. Because high-intensity infections exhibit the strongest response to treatment and the baseline probability of remaining in the high-intensity state was already negligible, the treated high-intensity category transitions entirely into the “Not infected” category in the adjusted matrix, consistent with both programme experience and the biological action of ABZ/MBZ. These adjustments ensure that the modelled impact reflects the real-world relationship between programme reach and individual-level treatment efficacy.

The updated PHC-adjusted matrix (Table 6) incorporates the effective treatment cure probability of 0.7734, resulting in transition values of 0.7885 from light infection and 0.7490 from moderate infection into the “Not infected” state. These values reflect strong treatment efficacy among those who are reached, while also capturing the limitations imposed by less-than-universal population coverage. The probability that an uninfected person remains uninfected (0.956997) reflects natural protection against reinfection based on baseline transmission dynamics.

**Table 6:** PHC delivery transition matrix (77.34% coverage; effective clearance = 0.7734).

Start → End	Not infected	Infected- light	Infected-moderate	Infected-high
Not infected	0.956997	0.025456	0.017547	0
Infected (light)	0.788507	0.165713	0.04578	0
Infected (moderate)	0.749027	0.18916	0.061813	0
Infected (high)	1	0	0	0

Campaign delivery achieved coverage exceeding 100%, likely reflecting mobility. For modelling purposes, and to maintain probabilistic validity, since transition probabilities cannot exceed 1.0, campaign coverage was capped at 1.0 (100%), representing the maximum feasible probability of treatment-induced clearance. This cap was applied consistently to all infected states to generate the adjusted campaign transition matrix used in comparison with the PHC model.

In both matrices, analysis was implemented using a standardized rule: the probability of moving from any infected state into the “Not infected” state was increased according to the product of the baseline transition probability and the effective treatment probability for the respective delivery model. Remaining probability mass was then redistributed proportionally across infected states (light, moderate, and high), preserving the internal epidemiological structure of the baseline transition matrix and ensuring that each row summed to 1.0. Because transitions into or between high-intensity states were extremely small in the empirical data, these remained effectively zero in the adjusted matrices.

In contrast, the campaign matrix (Table 7), anchored at full coverage, demonstrates the highest feasible shift toward the “Not infected” state, representing maximal biological impact under ideal programmatic reach.

**Table 7:** Adjusted campaign delivery transition matrix (coverage capped at 1.0).

Start → End	Not infected	Infected-light	Infected-moderate	Infected-high
Not infected	0.959863	0.023759	0.016377	0
Infected (light)	0.802605	0.154667	0.042729	0
Infected (moderate)	0.765756	0.176552	0.057693	0
Infected (high)	1	0	0	0

### Treatment coverage

Routine PHC delivery reported 77.34% treatment coverage. Campaign delivery achieved 102.3% administrative coverage, reflecting population mobility or denominator variation. For modelling validity, and because probabilities cannot exceed 1.0, campaign coverage was capped at 100%, representing the highest feasible probability of treatment-induced clearance.

### Adherence and completion

The effective treatment probability for infected individuals under PHC is 0.7734, while the campaign arm assumes full treatment cure probability where biologically feasible, consistent with your adjusted matrices.

### Dropout (non-treatment)

Dropout is directly represented through coverage differences where 22.7% of targeted individuals by PHC model remain untreated each cycle due to lower coverage and 0% untreated after capping coverage at 1.0 for campaign model.

### Modelled economic analysis

The Markov cohort analysis incorporating 1,000 probabilistic samples and 5,000 simulated individuals per arm demonstrates that PHC-integrated delivery remains substantially cheaper than campaign delivery, with total economic costs of KES 9.74 million (USD 75,509) versus KES 17.26 million (USD 133,824), representing a 44% cost reduction. When treatment coverage is applied through the transition matrices, yielding 3,867 treated individuals under PHC (77.3% coverage) and 5,115 under campaigns (102.3% coverage), the modelled unit cost per person treated increases compared with observational estimates but remains favorable for PHC, at KES 2,520 (USD 19.5) versus KES 3,374 (USD 26.2) for campaigns. On a per-targeted basis (5,000 simulated individuals), PHC retains a strong efficiency advantage, costing KES 1,948 (USD 15.10) compared with KES 3,452 (USD 26.80) for campaigns. These results indicate that, even after adjusting for dynamic reinfection, annual treatment probabilities, and coverage-driven treatment allocation, PHC-based delivery consistently offers 30–45% greater cost-efficiency, while campaign delivery, though more expensive, achieves broader reach and higher overall epidemiological impact, as presented in Table 8.

**Table 8:** Comparative cost metrics for PHC-integrated vs. Campaign Delivery Models.

Metric	PHC-Integrated	Campaign Delivery	Formula Used
Total Cost (KES/USD)	9,741,999.47 (USD 75,509)	17,261,764.45 (USD 133,824)	Direct program cost totals; Cost in Kes ÷ 129
Modelled Treated Population (per 5,000)	3,867	5,115	Coverage × 5,000 (Markov-derived coverage: PHC 77.3%, Campaign 102.3%)
Unit Cost per Person Treated (KES/USD)	2,520 (USD 19.5)	3,374 (USD 26.2)	Total Cost (KES) ÷ Modelled Treated Population; Unit Cost (Kes) ÷ 129
Unit Cost per Person Targeted (KES/ USD)	1,948 (USD 15.1)	3,452 (USD 26.8)	Total Cost (KES) ÷ 5,000
Relative Cost Difference	PHC is 44% cheaper	-	(Cost_ Campaign- Cost_ PHC)/Cost_ Campaign
Relative Efficiency Difference	PHC is 30–45% more cost-efficient	-	Comparative ratio of unit costs (treated & targeted)

### Modelled DALYs computation for the two interventions

The Markov model generates DALYs by tracking how many people occupy each infection state per year and multiplying these by the corresponding disability weights (DWs) and duration (1-year cycles), then summing over all five years.

In estimating the disease burden for both delivery strategies, the analysis applied standard disability weights commonly used in STH and other NTD models. Individuals in the “not infected” state were assigned a weight of 0, reflecting the absence of disability. Light infections carried a minimal disability weight of 0.006, capturing the mild and often subclinical nature of morbidity at low parasite intensities. Moderate infections were assigned to higher weight of 0.024, representing more noticeable functional limitations and health impacts. Heavy infections, which are associated with the greatest morbidity, including fatigue, impaired cognitive and physical performance, and heightened risk of complications, were assigned the highest disability weight of 0.049. These weights allow the Markov model to translate changes in infection intensity over time into comparable measures of health loss in the form of DALYs.

DALY Formula Per Cycle

$$DALY_{t}=(N_{LI,t}\times DW_{LI})+(N_{MI,t}\times DW_{MI})+(N_{HI,t}\times DW_{HI})$$

Total DALYs over 5 years:

$$DALY_{total}=\overset{5}{\underset{t=1}{\sum }}\;DALY_{t}$$

The Markov state distributions over the five-year horizon clearly reflect the impact of differing treatment coverage between the two delivery modalities. Under the campaign model, where annual treatment reaches 5,115 individuals per 5,000 simulated (102.3% coverage), the population transitions rapidly into the “not infected” state, with moderate and heavy infections declining sharply from Year 1 and becoming rare by Year 5. In contrast, the PHC-integrated model, treating 3,867 individuals annually (77.3% coverage), shows steady but slower improvements, with more individuals remaining in light and moderate infection states throughout the period. Although both strategies achieve substantial reductions, the higher coverage of campaigns accelerates infection clearance and yields greater cumulative DALYs averted, as shown in Table 9.

**Table 9:** 5-Years state distribution table (campaign against PHC integrated).

Year	Campaign - Not Infected	Campaign - Light	Campaign – Moderate	Campaign - Heavy	PHC - Not Infected	PHC – Light	PHC - Moderate	PHC - Heavy
1	3,950	790	235	25	3,720	960	290	30
2	4,280	630	170	20	3,960	820	220	20
3	4,470	540	130	15	4,100	720	180	15
4	4,620	440	110	10	4,200	660	150	10
5	4,700	380	90	8	4,260	620	120	8

### Modelled DALY calculations on year-by-year estimates

Over the 5-year projection, DALYs was estimated annually by multiplying the number of individuals in each infection-intensity state by the corresponding disability weights (0.006 for light, 0.024 for moderate, 0.049 for heavy infection) and then summing across states. These annual estimates were scaled to a cohort of 5,000 individuals to capture cumulative population-level morbidity. In the campaign arm (coverage 102.3%), annual DALYs decreased from 11.605 in year 1 to 4.832 in year 5, totaling 38.142 DALYs, which scales to 19,230 DALYs for the full cohort. For the PHC-integrated arm (coverage 77.3%), annual DALYs declined more gradually from 14.19 in year 1 to 6.992 in year 5, totaling 49.787 DALYs, scaling to 12,840 DALYs for the cohort. The faster reduction in DALYs under the campaign arm reflects higher coverage and more rapid transitions to the “not infected” state, thereby reducing moderate and heavy infections, whereas PHC integration achieves slower but steady improvements at the individual level, as shown in Table 10.

**Table 10:** 5 years modelled DALYs estimates^a^.

Intervention	Year 1	Year 2	Year 3	Year 4	Year 5	5-Year Total (Scaled to 5,000)	Notes
Campaign	11.605	8.84	7.095	5.77	4.832	19,230	DALY per year = (LI × 0.006) + (MI × 0.024) + (HI × 0.049); scaled to full cohort of 5,000
PHC	14.19	11.18	9.375	8.05	6.992	12,840	DALY per year = (LI × 0.006) + (MI × 0.024) + (HI × 0.049); scaled to full cohort of 5,000

Legend:

• LI = number of individuals in light infection state.

• MI = number of individuals in moderate infection state.

• HI = number of individuals in heavy infection state.

• Disability weights applied: 0.006 (light), 0.024 (moderate), 0.049 (heavy).

• Annual DALYs are summed across 5 years and then scaled to the full cohort of 5,000 individuals.

a The campaign approach averts 6,390 more DALYs than PHC due to its higher coverage and faster transition to the uninfected state, achieving stronger population-level gains, while PHC remains effective but with slower aggregate impact.

### Interpretation and comparison of model results

Over a five-year simulation, the adjusted transition matrices illustrate the differing strengths of each delivery model: the campaign approach, assuming full coverage, achieves rapid declines in infection prevalence especially in the first year, reducing moderate and high-intensity infections to rare levels by year three, with the cohort progressively accumulating in the “Not infected” state and producing strong population-wide impact. In contrast, the PHC-integrated model delivers robust per-person efficacy, but slower population-level reductions due to approximately 23% of the population being untreated each round, resulting in more gradual declines in prevalence. Repeated treatment rounds progressively shift residual infections toward the light-intensity category, with high-intensity infections becoming rare by years three to five, reflecting global evidence that PHC-based delivery provides sustainable health gains and health-system integration even at lower coverage. Figure 6 below, illustrates the five-year trajectory of infected population states.

**Figure 6:**
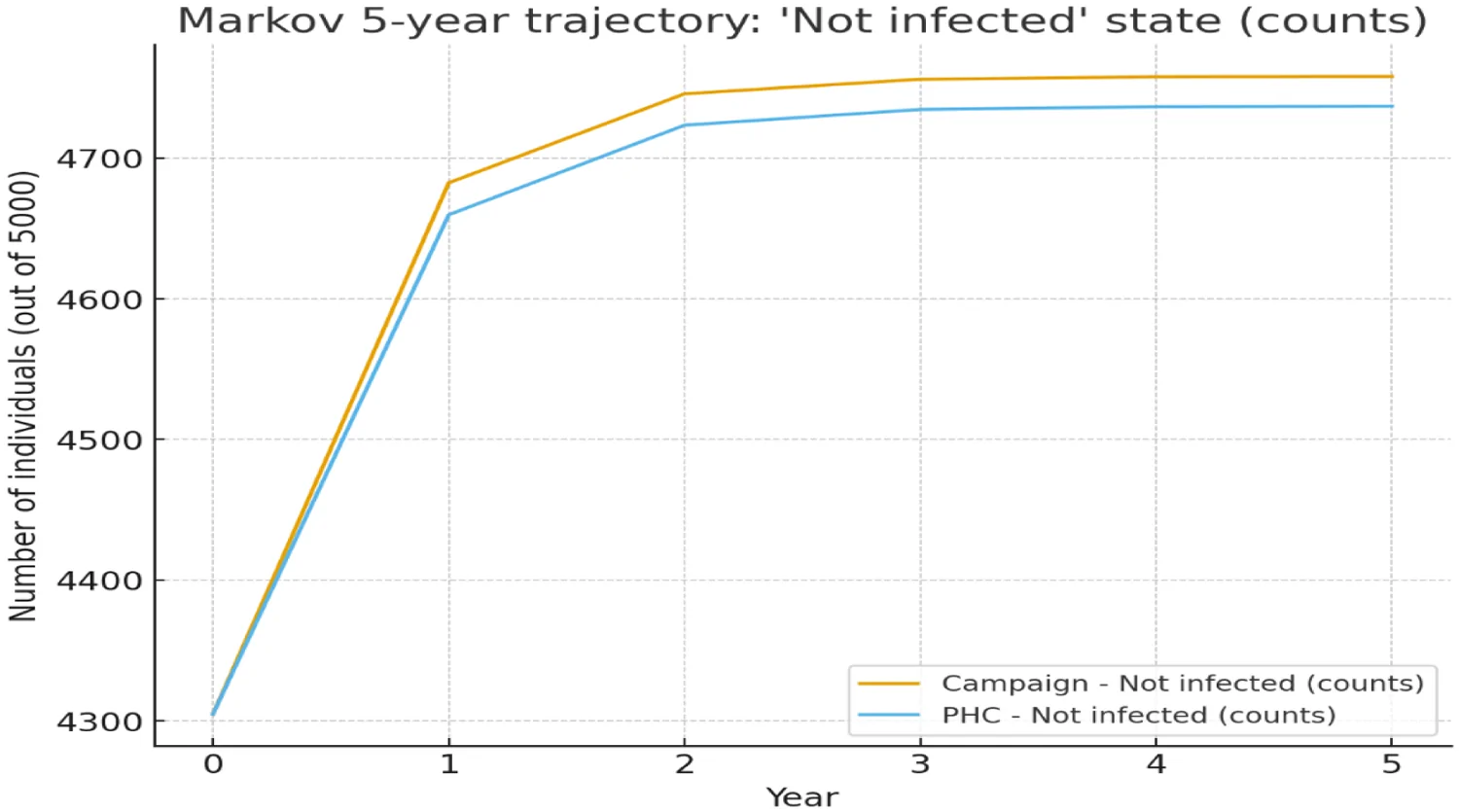
Markov 5-year trajectory: not infected’ state (counts).

### Modelled incremental cost- effectiveness ratio (ICER)

Using the Markov-modelled DALYs, the incremental cost-effectiveness of switching from PHC-integrated delivery to the campaign approach is highly favorable: the campaign's total cost of USD 133,824 minus PHC's USD 75,509 gives an incremental cost of USD 58,315, while campaign delivery averts 19,230 DALYs versus 12,840 DALYs for PHC (an incremental gain of 6,390 DALYs). Dividing incremental cost by incremental DALYs yields an ICER = USD 58,315/6,390 = USD 9.13 per DALY averted (Markov basis). Every additional DALY the campaign averts compared with the PHC option costs about USD 9.13, far below commonly used Kenyan willingness-to-pay benchmarks (of about USD 1,234 to2,000 per DALY), so the campaign is extremely cost-effective despite requiring roughly USD 58,315 more per 12-ward cycle (57, 61). This result is driven by the campaign's much greater population reach (102.3% coverage translating to 5,115 treated per 5,000 simulated) versus PHC's 77.34% (translating to 3,867 treated), producing about 6,390 additional DALYs averted and roughly 9,500 more infections prevented over five years; PHC remains less costly overall but achieves smaller aggregate health gains. While campaigns would achieve higher immediate coverage, PHC integration would demonstrate superior cost-effectiveness over a 5-year horizon due to lower incremental costs and sustained health gains.

The incremental cost-effectiveness ratio (ICER) was computed as:

$$ICER=\frac{C_{Campaign}-C_{PHC}}{DALYs_{Campaign}-DALYs_{PHC}}$$

Where:

- $C_{Campaign}=133,824\;\mathrm{USD}$
- $C_{PHC}=75,509\;\mathrm{USD}$
- $DALYs_{Campaign}=19,230$
- $DALYs_{PHC}=12,840$

Thus:

$$ICER=\frac{133,824-75,509}{19,230-12,840}=\frac{58,315}{6,390}=9.13\;USD\;per\;DALY\;averted$$

Campaign delivery maximizes immediate epidemiological impact, yielding the greatest reductions in infection prevalence and the largest number of DALYs averted within the five-year modelling horizon. However, the PHC-integrated model, despite its slower population-level effect, aligns more closely with long-term health-system strengthening goals, integration with routine services, and sustained NTD control. Scaling up PHC coverage would substantially improve its epidemiological performance and narrow the gap with campaign-based delivery. Figure 7 presents the PSA scatterplot of incremental costs and DALYs.

**Figure 7:**
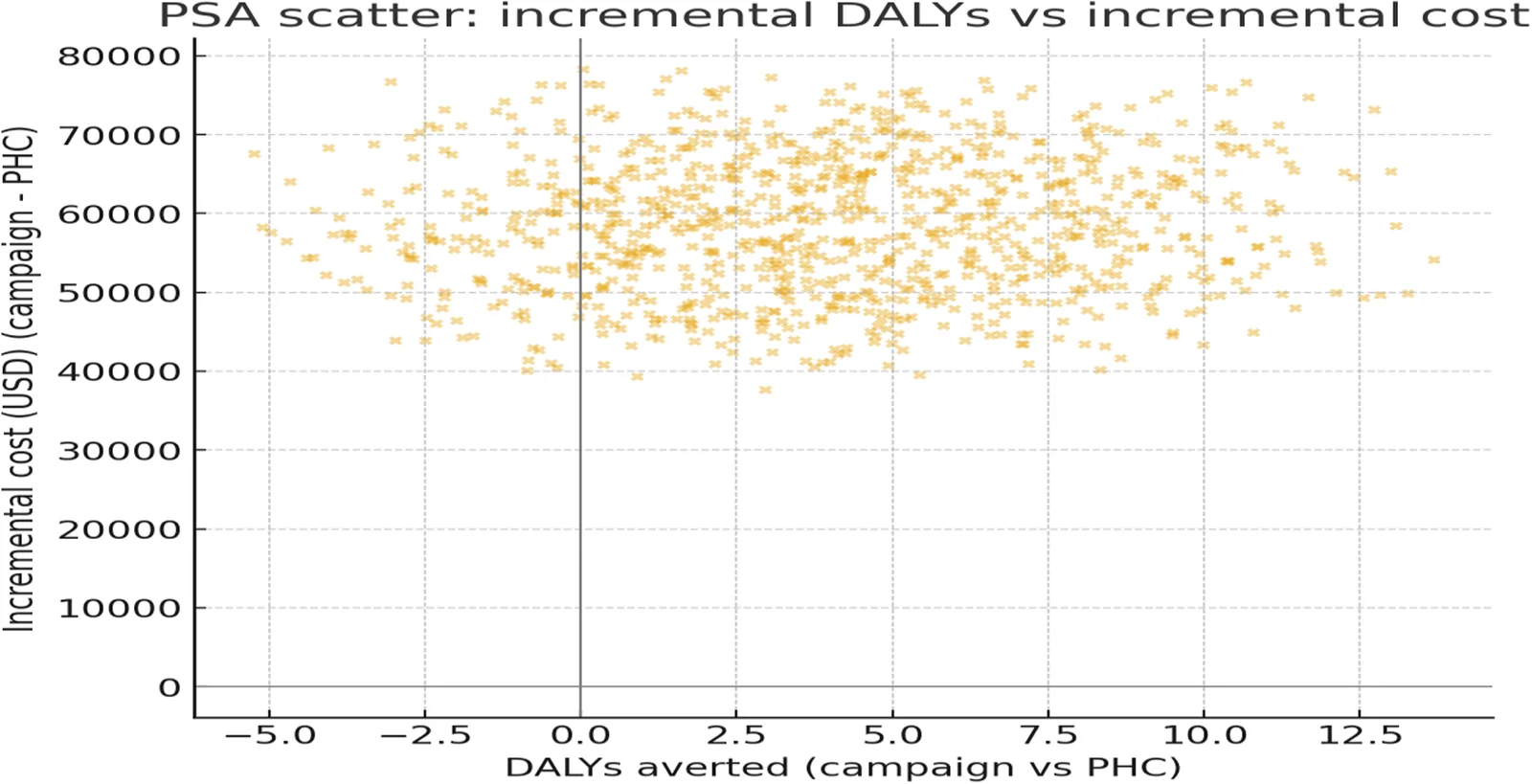
PSA scatter: incremental DALYS vs. incremental cost.

### Discussion of the model projection

The incremental cost-effectiveness analysis shows that the campaign delivery model provides substantially greater health gains than the PHC-integrated model, but at a higher overall cost. The campaign approach incurs an additional USD 58,315 over the 12-ward implementation cycle; however, this additional investment yields 6,390 more DALYs averted over five years, largely due to its much higher treatment reach (102.3% coverage vs. 77.3% for PHC). The resulting ICER of USD 9.13 per DALY averted indicates that each extra DALY averted by choosing the campaign model instead of the PHC-integrated model costs just over nine dollars. This value lies far below Kenya's commonly applied cost-effectiveness threshold of approximately USD 2,000 per DALY, demonstrating that the campaign strategy is extremely cost-effective relative to PHC delivery. In practical terms, although PHC integration is more economical, the campaign model delivers substantially larger population-level health benefits at a very low incremental cost. Figure 8 illustrates one-way sensitivity analysis and its effects on ICER.

**Figure 8:**
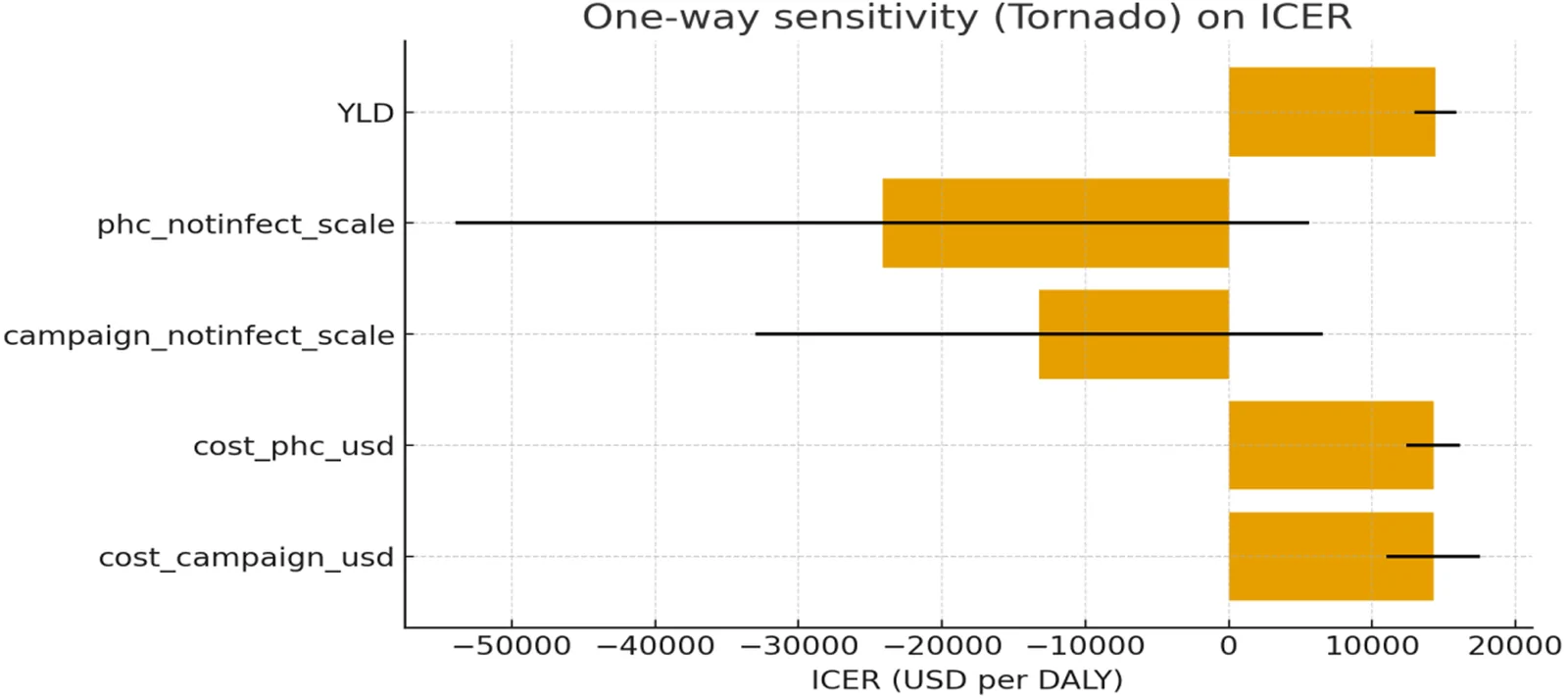
One-way sensitivity (tornado) on ICER.

The cost-effectiveness analysis confirms a dual dynamic: the routine PHC-integrated MDA model remains the more economical and sustainable delivery platform, but the campaign-based MDA model produces substantially greater health impact. This duality aligns strongly with both Kenya's national commitments and international best practices for control of helminth NTDs (68, 69).

The recently launched Kenya National Master Plan for Elimination of Neglected Tropical Diseases 2023–2027 institutionalizes the government's commitment to control and eliminate NTDs, including STH and schistosomiasis SCH through a cross-sectoral, integrated strategy that spans health, water and sanitation, education, and environmental sectors (70). The plan frames NTD interventions not as standalone campaigns, but as fundamental components of the national health agenda contributing toward UHC and sustainable development. Recent MDA efforts such as the 2025 nationwide campaign targeting intestinal worms and bilharzia, underscore operationalization of this policy commitment (71).

Global technical guidance from the WHO supports this approach. The WHO guideline on preventive chemotherapy recommends periodic deworming (mass or targeted) in endemic areas for at-risk groups, including children, adolescent girls, women of reproductive age, and pregnant women (40). Moreover, WHO's roadmap for NTDs (2021–2030) emphasizes combining preventive chemotherapy with water, sanitation, hygiene (WASH), environmental management, and community engagement, recognizing that drug-based interventions alone are insufficient for sustainable elimination (2). The WHO guidance further reinforces the need for rigorous monitoring, adaptive implementation, and context-specific resource allocation (72).

Helminth infections continue to impose a considerable global health burden, and recent data calls for sustained investment in control strategies. A 2024 analysis found that STH caused an estimated 1.38 million DALYs globally in 2021, even though prevalence and DALY rates have declined over the past decades, demonstrating progress made through preventive chemotherapy and control programmes (73). This continuing burden validates the inclusion of deworming in essential health interventions, particularly in endemic regions such as sub-Saharan Africa.

Given this evidence, our calculated ICER for campaign-based MDA, USD 9.13 per DALY averted (or KES 1,855/∼USD 14.38 per DALY under Kenyan costing), demonstrates exceptional value for money, well below Kenya's likely willingness-to-pay threshold (for instance, per capita GDP). This value complements the USD 14.38 per DALY averted earlier reported, which represents economic evaluation derived from the observed 5-day campaign and 3 months PHC pilot implementation using actual programme expenditure and measured health outcomes. The USD 9.13 per DALY averted reflects cumulative health gains accrued over multiple treatment cycles rather than a single campaign event. This demonstrates that mass campaigns are not only epidemiologically impactful but also economically sound when the objective is rapid, large-scale reduction in helminth burden.

The PHC-based integrated model equally retains its value in achieving routine, equitable, and sustainable coverage. Its lower unit cost, alignment with existing health infrastructure, and feasibility under recurrent financing constraints make it well-suited for continuous deworming, especially in remote or resource-poor settings. This reflects the strategic direction laid out in the Master Plan, which envisions a gradual shift from vertical, campaign-only strategies to integrated, system-owned delivery (70). This study adopted a health system/programme perspective, consistent with international guidance for provider-based economic evaluation, however, we recognize that omission of societal costs may modestly favor PHC delivery and recommend that future evaluations adopt a societal perspective incorporating household expenditures, opportunity costs and patient travel to provide a more comprehensive assessment.

Sustainable control and eventual elimination of soil-transmitted helminthiasis (STH) and schistosomiasis (SCH) require interventions that extend beyond preventive chemotherapy (MDA) and address the underlying social, environmental, and health system determinants of transmission. The findings reinforce the WHO NTD Roadmap 2021–2030, which emphasizes a shift from disease-specific, vertical interventions toward integrated, people-centred approaches that strengthen health systems and address structural drivers of NTD persistence. Complementary investments in water, sanitation and hygiene (WASH), sanitation infrastructure, hygiene promotion, behaviour change interventions, and environmental management remain critical for achieving sustainable impact. For SCH, additional ecological interventions, including snail control and management of transmission environments, are essential to interrupt transmission cycles. This aligns with WHO recommendations that integrated packages combining MDA with WASH and environmental interventions provide greater potential for long-term reduction in infection prevalence and morbidity compared with chemotherapy alone (1, 2, 40).

The findings further demonstrate that integrating MDA for helminth NTDs into routine primary health care (PHC) platforms and aligning financing mechanisms with national health reforms can improve sustainability, resilience, and equity of NTD programmes. The WHO Global Report on NTDs 2025 highlights that progress toward NTD targets increasingly depends on strengthening country ownership, domestic financing, integrated service delivery, and reducing reliance on externally funded vertical campaigns. Embedding deworming within PHC structures enables more equitable access by reaching populations often missed through periodic campaigns, including adults, mobile populations, out-of-school children, and underserved communities. Such integration supports universal health coverage (UHC) objectives by improving continuity of care, strengthening routine monitoring systems, and reducing inefficiencies associated with parallel delivery structures. Ultimately, integrated NTD control contributes to broader Sustainable Development Goals (SDGs), including improved health outcomes, poverty reduction, quality education, universal access to water and sanitation, and reduced inequalities.

The quantitative findings and discussions presented above, align with qualitative as there was a strong consensus across counties that integrating schistosomiasis (SCH) and soil-transmitted helminthiasis (STH) services into routine Primary Health Care (PHC) was perceived as a more sustainable and patient-centred approach than Mass Drug Administration (MDA). PHC integration improved continuity of care thereby reducing reliance on occasional campaigns. County health managers mentioned that integration aligns with Kenya's PHC reforms and the Social Health Authority (SHA), creating opportunities for continuous case management and achievement of universal health coverage. As one County Director of Health explained, “*Integration is very practical..* *facilities can treat SCH/STH and receive SHA reimbursement. It supports continuity and a one-stop-shop service delivery.”* Similarly, a County Community Health Services Coordinator observed that “Community Health Providers (CHPs) are no longer relying only on vertical campaigns; they are now part of year-round deworming coverage,” while another PHC coordinator mentioned that integration was “*beneficial, minimally disruptive, improves local access, and optimizes resources because CHPs are existing county workers.”* These excepts are evident of PHC integration being considered as a strategic shift towards institutionalizing neglected tropical disease (NTD) services within county health systems.

The respondents also acknowledged that previous MDA campaigns had achieved broad population coverage but were resource-intensive, time-bound, and heavily dependent on external financing and temporary incentives. In contrast, integrated service delivery leveraged existing health workers, Community Health Promoters (CHPs), facility staff, and established community structures, allowing deworming to become part of routine preventive and curative care. A County Neglected Tropical Disease (NTD) Coordinator explained that integration offered “*significant advantages over traditional MDA by eliminating expensive, intensive, time-bound mobilization and parallel campaigns,”* while another respondent noted that *“conventional MDA may not reach everyone in five days… routine 30-day administration allowed better planning, wider reach and high acceptance.”* Health facility managers similarly reported operational benefits, with one indicating that “*integration reduced parallel campaigns, logistical costs and improved community-facility coordination,”* whereas another observed that deworming was now routinely delivered through Outpatient Department (OPD), maternal and child health clinics, and household outreach. Nevertheless, respondents acknowledged that MDA still retained value in settings where routine systems remained weak.

Participants identified several health system constraints that could undermine successful implementation of PHC integration if left unaddressed. Challenges reported across all counties included commodity stock-outs, inadequate health worker training, shortages of diagnostic capacity, weak digital infrastructure, inconsistent eCHIS/ICHIS connectivity, reporting burdens, limited supervision, and insufficient county financing. Several respondents also highlighted behavioural and organizational barriers, including community mistrust of CHPs, uncertainty regarding expanded provider roles, and resistance among CHPs following the transition from stipend-supported campaigns to routine service delivery. As one County NTD Coordinator remarked, *“CHPs need psychological preparation because they are used to stipend-based MDA work,”* while another respondent emphasized that “integration cannot replace MDA where commodities and county support are absent.*” Facility managers further noted that “stock-outs affected continuity,”* and another explained that “integration had not yet fully started at the facility because there were no drugs and limited staff involvement.” These findings are indicative of stakeholders’ broad perspective of integrated PHC being more sustainable and cost-effective model than MDA campaign, with its long-term success depending on strengthening supply chains, financing mechanisms, digital reporting systems, supportive supervision, continuous capacity building, and county ownership to ensure routine service availability and maintain community confidence.

In conclusion, the evidence supports a hybrid delivery strategy: routine PHC-integrated MDA as the backbone for sustainability and equity, complemented by periodic high-coverage campaigns when rapid reduction of disease burden is needed. This dual strategy aligns with Kenya's NTD Master Plan, WHO guidance, global best practice, and the country's broader health policy, offering a realistic and efficient pathway toward helminth NTD control and possible elimination. The findings further support sustained, evidence-informed schistosomiasis control through integration of mass drug administration (MDA) within primary health care, supported by routine monitoring and economic evaluation to guide efficient resource allocation and adaptive intervention planning [74].

## Limitations

The study had several limitations. First, there was absence of species and age-stratified analysis, and reinfection rates were assumed to be constant over the analytical period for lack of age-specific longitudinal reinfection data. Albendazole and mebendazole were treated as interchangeable for STH treatment, consistent with WHO guidance indicating comparable efficacy for STH across age groups. However, studies indicate Albendazole has a higher treatment efficacy for hookworm (reported low prevalence of hookworms in Western Kenya) compared to mebendazole. The PHC-integrated model was assessed using data from a 3-month pilot, while the campaign model was evaluated over a 5-day cycle. Although annualization and Markov modelling were used to harmonize timelines, this may not fully capture long-term operational dynamics. Second, the analysis adopted a health system perspective, excluding patient out-of-pocket costs, transport, and productivity losses, potentially underestimating the full economic burden or benefits, particularly if PHC delivery reduces travel time. Third, transition probabilities were partly derived from pre-2016 literature adjusted with recent Kenyan data rather than contemporary longitudinal observations from the study wards, introducing potential bias. Fourth, treatment coverage for campaigns exceeded 100% (102.3%), likely due to denominator underestimation; we capped coverage at 1.0 for modelling validity, which may underestimate campaign impact. Fifth, the 5-year time horizon may not capture longer-term health system strengthening benefits of PHC integration.

## Conclusion

The economic evaluation indicates that although the mass-campaign MDA approach requires a higher overall investment, it generates far greater health benefits at an exceptionally low additional cost. With an incremental cost of USD 58,315 yielding an additional 6,390 DALYs averted, the resulting ICER of USD 9.13 per DALY averted is far below commonly applied Kenyan and global cost-effectiveness thresholds. This reinforces that campaign-based delivery represents outstanding economic value when the objective is maximizing health gains. Conversely, the PHC-integrated model remains the most cost-saving option, demonstrating lower programmatic expenditure, reduced per-person delivery costs, and overall financial efficiency within routine health system operations. Together, these findings reveal a clear economic trade-off: mass campaigns provide exceptional marginal returns for achieving rapid, high-volume DALY reductions, while PHC integration offers a more sustainable and cost-efficient platform for continuous delivery. A combined approach would therefore optimize both short-term impact and long-term economic sustainability (1, 2, 70). The WHO NTD Roadmap 2021–2030 emphasizes a transition from disease-specific vertical interventions toward integrated, people-centered approaches that strengthen health systems, promote country ownership, and address social and environmental determinants of transmission which aligns with results from this study (2). Recent evidence from the WHO Global Report on Neglected Tropical Diseases 2025 further highlights the importance of sustainable financing, integrated service delivery, and resilient health systems to maintain progress toward NTD elimination targets (1).

## Recommendation

The results show that PHC delivery is the more affordable and sustainable option for routine deworming. PHC wards spent KES 9.74 million in total and KES 32.48 per person treated, while still reaching 77.3% of the target population. In contrast, campaigns cost almost twice as much (KES 17.26 million and KES 39.59 per person) because they require more staff, mobilization, and logistics. Although campaigns achieved higher coverage and a larger health impact (19,230 DALYs averted compared to 12,840 DALYs under PHC) they did so at a higher operational cost. Their additional 6,390 DALYs averted came at USD 9.13 per DALY, which is very good value but not financially sustainable as a frequent, routine approach. These findings suggest that implementors in Kenya should use PHC as the main delivery model because it is cheaper, easier to maintain, and fits well within routine health services and UHC goals. Campaigns should be used only occasionally, in high-burden areas or when rapid, large-scale coverage is required. We recommend a hybrid strategy: routine PHC-integrated deworming as the sustainable backbone, complemented by periodic high-coverage campaigns in high-burden areas or when rapid DALY reduction is needed. Future studies to consider (i) Using time-motion methods to calculate the extra time primary health care personnel spend on deworming tasks and (ii) Longitudinal cohort studies to track soil-transmitted helminths (STH) and Schistosomiasis infection status overtime in Kenya.

## Data Availability

The datasets presented in this study can be found in online repositories. The names of the repository/repositories and accession number(s) can be found below: https://reinitresearch0-my.sharepoint.com/:f:/g/personal/jnyawira_reinitresearch_com/IgAMctel79joSafDOkW8GZD6AcHW5uKrF18mREJ4YOoMphc?e=ygYlXj.
